# BRAF inhibition sensitizes melanoma cells to α-amanitin via decreased RNA polymerase II assembly

**DOI:** 10.1038/s41598-019-44112-7

**Published:** 2019-05-23

**Authors:** Lukas Frischknecht, Christian Britschgi, Patricia Galliker, Yann Christinat, Anton Vichalkovski, Matthias Gstaiger, Werner J. Kovacs, Wilhelm Krek

**Affiliations:** 1 Institute of Molecular Health Sciences, ETH Zurich, 8093 Zurich, Switzerland; 2 Institute of Molecular Systems Biology, ETH Zurich, 8093 Zurich, Switzerland; 3 Present Address: Department of Medical Oncology and Hematology, University Hospital of Zurich and University of Zurich, 8091 Zurich, Switzerland

**Keywords:** Melanoma, Targeted therapies

## Abstract

Despite the great success of small molecule inhibitors in the treatment of patients with BRAF^V600E^ mutated melanoma, the response to these drugs remains transient and patients eventually relapse within a few months, highlighting the need to develop novel combination therapies based on the understanding of the molecular changes induced by BRAF^V600E^ inhibitors. The acute inhibition of oncogenic signaling can rewire entire cellular signaling pathways and thereby create novel cancer cell vulnerabilities. Here, we demonstrate that inhibition of BRAF^V600E^ oncogenic signaling in melanoma cell lines leads to destabilization of the large subunit of RNA polymerase II POLR2A (polymerase RNA II DNA-directed polypeptide A), thereby preventing its binding to the unconventional prefoldin RPB5 interactor (URI1) chaperone complex and the successful assembly of RNA polymerase II holoenzymes. Furthermore, in melanoma cell lines treated with mitogen-activated protein kinase (MAPK) inhibitors, α-amanitin, a specific and irreversible inhibitor of RNA polymerase II, induced massive apoptosis. Pre-treatment of melanoma cell lines with MAPK inhibitors significantly reduced IC50 values to α-amanitin, creating a state of collateral vulnerability similar to *POLR2A* hemizygous deletions. Thus, the development of melanoma specific α-amanitin antibody-drug conjugates could represent an interesting therapeutic approach for combination therapies with BRAF^V600E^ inhibitors.

## Introduction

Melanoma is an aggressive tumor with poor survival when disseminated to distant organs. In recent years, two main types of treatment have been developed for melanoma, which substantially helped to increase overall survival of patients: immune checkpoint inhibitors and targeted therapies^1^. BRAF-mutated metastatic melanoma has become a prototype for therapies targeting oncogenic activated signaling cascades. Several compounds inhibiting BRAF^V600E^ and the downstream mitogen-activated protein kinase (MAPK) signal transduction pathway were developed and are now successfully used in the clinic^2^. However, these therapies are effective in only a subset of patients, approximately 50% with tumors harboring the gain-of-function BRAF^V600E^ mutation, and drug resistance inevitably develops and most patients relapse within a few months^1,2^.

The acute inhibition of oncogenic driver mutations by small molecule inhibitors can rewire entire signaling cascades within hours and might thereby increase the dependency on certain cellular functions^3^. A better mechanistic understanding of the acute response of cancer cells to inhibition of oncogenic signaling as well as their response to diverse intra- or extracellular stresses will help to elucidate novel cancer cell vulnerabilities and to develop combination therapies^3–5^.

Chaperone proteins are considered to be central nodes in cellular homeostasis. Through their transient interactions with diverse client proteins they are involved in several signaling pathways. Posttranslational modifications of the chaperone proteins themselves or of client proteins by oncogenic signaling can influence their interactions and thereby change the signaling output^6–9^. The unconventional prefoldin RPB5-interactor (URI1) is a member of the prefoldin family of evolutionary conserved molecular chaperones. It was shown to be a direct phosphorylation target of p70-S6 kinase 1 (S6K1). This URI1 phosphorylation is controlling its interaction with the phosphatase PP1γ (protein phosphatase 1, catalytic subunit, gamma isoform) and thereby PP1γ-mediated de-phosphorylation of S6K1^8^. Furthermore, it was demonstrated that *URI1* is amplified and has oncogenic functions in ovarian cancer by sequestration of PP1γ in an inactive complex, thus preventing the PP1γ-mediated negative feedback and shifting the apoptotic balance towards increased survival^10^. In addition, a subset of *URI1* non-amplified colorectal cancer (CRC) cell lines has developed a strong dependency on the URI1 chaperone system^11^.

URI1 is an evolutionary conserved interactor of POLR2E (polymerase RNA II DNA-directed polypeptide E, also known as RPB5), a shared subunit of all three RNA polymerases^12–14^. Together with its co-members of the URI1/‘prefoldin-like’ and the R2TP [RUVBL1, RUVBL2, RPAP3 (in yeast known as Tah1), PIH1D1] complex it is tightly involved in the cytoplasmic assembly of all three RNA polymerase complexes^13,15–17^.

Recently, transcription and RNA polymerases have become interesting novel therapeutic targets^18,19^. In 2015 Liu *et al*.^18^ showed that colorectal cancer cell lines with only one allele of *POLR2A* (polymerase RNA II DNA-directed polypeptide A) and therefore lower POLR2A protein levels demonstrate an increased sensitivity to α-amanitin, a selective and specific inhibitor of RNA polymerase II. Furthermore, they showed that *POLR2A* as a flanking gene of the tumor suppressor gene *TP53* on chromosome 17p, is co-deleted in approximately half of CRC cases^18^. α-amanitin, a cyclic peptide of eight amino acids, is a toxin found in the mushroom *Amanita phalloides*. In contrast to other transcription inhibitors, it binds to POLR2A with very high affinity, causes a transcriptional arrest and leads to the degradation of POLR2A^18,20,21^. To circumvent its liver toxicity Liu *et al*. constructed an anti-epithelial cell adhesion molecule (EpCAM) antibody coupled to α-amanitin. Using this antibody-drug conjugate they were able to show complete tumor regressions in mouse models of human colorectal cancer with a hemizygous deletion of *POLR2A*^18^.

Given the strong dependency of several cancer cells throughout all cancer types on the URI1 chaperone complex, its critical involvement in the RNA polymerase assembly and the direct influence of oncogenic signaling on URI1 interaction with client proteins, we investigated the effect of acute inhibition of BRAF^V600E^ oncogenic signaling on the URI1 interactome in melanoma cells.

## Results

### Inhibition of oncogenic BRAF^V600E^ decreases interaction of RNA polymerase II subunits with the chaperone URI1 in melanoma cell lines

**Figure 1:**
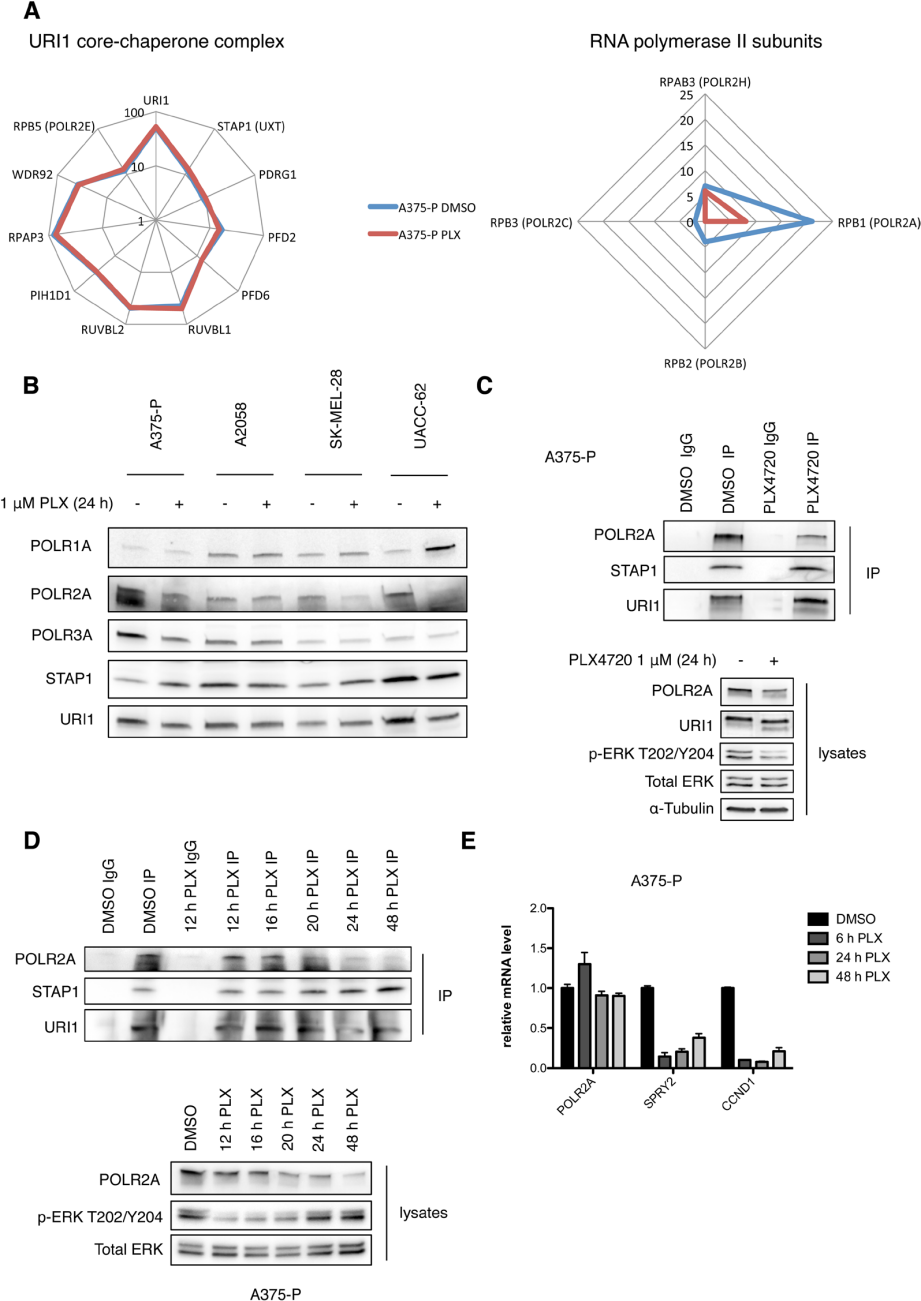
Inhibition of oncogenic BRAF^V600E^ decreases interaction of RNA polymerase II subunits with the chaperone URI1 in melanoma cell lines. (**A**) Mass-spectrometric analysis of URI1 interaction partners in A375-P cells treated for 16 hours with 1 μM PLX4720 (PLX) (red) or DMSO control (blue). Shown are the spectral counts of the URI1 core chaperone complex (logarithmic scale) and of the four subunits of RNA polymerase II, that were detected (linear scale). (**B**) Representative immunoblot showing URI1 co-immunoprecipitation of A375-P, A2058, SK-MEL-28 and UACC-62 cells treated with PLX4720 or DMSO as control for 24 hours. (**C**) Representative URI1 co-immunoprecipitation of A375-P cells treated with 1 μM of the BRAF^V600E^ inhibitor PLX4720. The lower panel shows the respective input lysates. (**D**) Representative URI1 co-immunoprecipitation of A375-P cells treated for the indicated time points with 1 μM PLX4720. The lower panel shows the respective input lysates, p-ERK and total ERK served as a treatment and loading control (n = 3). (**D**) Expression of *POLR2A* and the ERK target genes *SPRY2* and *CCND1* after indicated time of PLX4720 treatment in A375-P cells. Each value represents the amount of mRNA relative to that in DMSO-treated cells, which was arbitrarily defined as 1. Data are mean ± SD (n = 3 biological replicates).

We performed a mass spectrometry analysis to investigate whether BRAF^V600E^ oncogenic signaling might influence interactions of the URI1 chaperone complex with client proteins in melanoma cell lines. For this purpose the BRAF^V600E^-mutated melanoma cell line A375-P was treated with 1 μM of the vemurafenib analogue PLX4720 for 16 h, followed by URI1 immunoprecipitation and proteomics analysis. The PLX4720 concentration was chosen based on the data of the original description of PLX4720 as well as experience in our lab showing the best phospho-ERK suppression without induction of massive apoptosis^22^. Supplementary Table 1 shows all URI1 interactors with their respective spectral counts detected in our proteomics analysis. Apart from the members of the URI1/‘prefoldin-like’ and the R2TP chaperone complexes, we detected several subunits of the three nuclear RNA polymerases, as well as other transcription regulatory proteins like the PAF1 (Polymerase-Associated Factor 1) complex. Furthermore, all three isoforms of protein phosphatase 1 were detected. Acute inhibition of BRAF^V600E^ did not affect the composition of the URI1 core-chaperone complex (*i.e*., the ‘prefoldin-like’ and R2TP complex members: URI1, STAP1, PDRG1, PFD2, PFD6, RUVBL1, RUVBL2, RPAP3, PIH1D1, WDR92 and POLR2E) nor its association with transcription regulatory proteins. However, the interaction with most RNA polymerase subunits that were detected strongly decreased upon PLX4720 treatment. This was especially prominent for the subunits of RNA polymerase II (Fig. 1A and Supplementary Table 1). Furthermore, the gamma isoform of protein phosphatase 1 only bound to URI1 when BRAF^V600E^ signaling was inhibited (Supplementary Table 1), similar as already described for PP1γ binding to URI1 when S6K1 signaling is inhibited^8^. To confirm the observed effect of BRAF^V600E^ inhibition on URI1 interaction with RNA polymerases, we performed URI1 co-immunoprecipitation experiments in a panel of BRAF^V600E^-mutated melanoma cell lines (A375-P, A2058, SK-MEL-28 and UACC-62) treated with PLX4720 for 24 h and blotted for each of the large subunits of the three nuclear RNA polymerases POLR1A, POLR2A and POLR3A [polymerase RNA (I, II, III) DNA-directed polypeptide A]. The large subunits thereby served as a representative of the corresponding RNA polymerase complex. Only URI1 interaction with POLR2A consistently decreased in three out of four melanoma cell lines (Fig. 1B). Interaction with STAP1 (SKP2-Associated Alpha PFD 1), a member of the URI1/‘prefoldin-like’ complex, was not changed in any of the cell lines upon BRAF^V600E^ inhibition (Fig. 1B). This finding was confirmed in A375-P and UACC-62 cell lines with the MEK1/2 inhibitor AZD6244, which showed similar results in both cell lines as we observed with PLX4720 (Figs 1C, S1B and S2). To further assess the dynamics of URI1/POLR2A interaction upon BRAF^V600E^ inhibition, we treated A375-P cells for 12, 16, 20, 24 and 48 h with PLX4720. A steady decrease of POLR2A binding to URI1 was observed (Fig. 1D). This decreased interaction was accompanied by a reduction in POLR2A protein levels in PLX4720-treated cells, which occurred concomitantly with the decrease of URI1/POLR2A complex abundance (Fig. 1D). The well-known reactivation of ERK-signaling after prolonged PLX4720 treatment^23,24^ did not lead to an up-regulation of POLR2A protein levels or increase of URI1/POLR2A complexes (Fig. 1D). Furthermore, *POLR2A* mRNA expression in A375-P and UACC-62 cells did not change upon PLX4720 treatment, thereby excluding a direct effect of inhibiting the ERK-signaling pathway on *POLR2A* transcription, whereas mRNA levels of the ERK target genes *SPRY2* (sprouty homolog 2) and *CCND1* (cyclin D1) were decreased in PLX4720-treated cells (Figs 1E and S2B).

### POLR2A is destabilized by inhibition of oncogenic BRAF^V600E^ signaling

**Figure 2:**
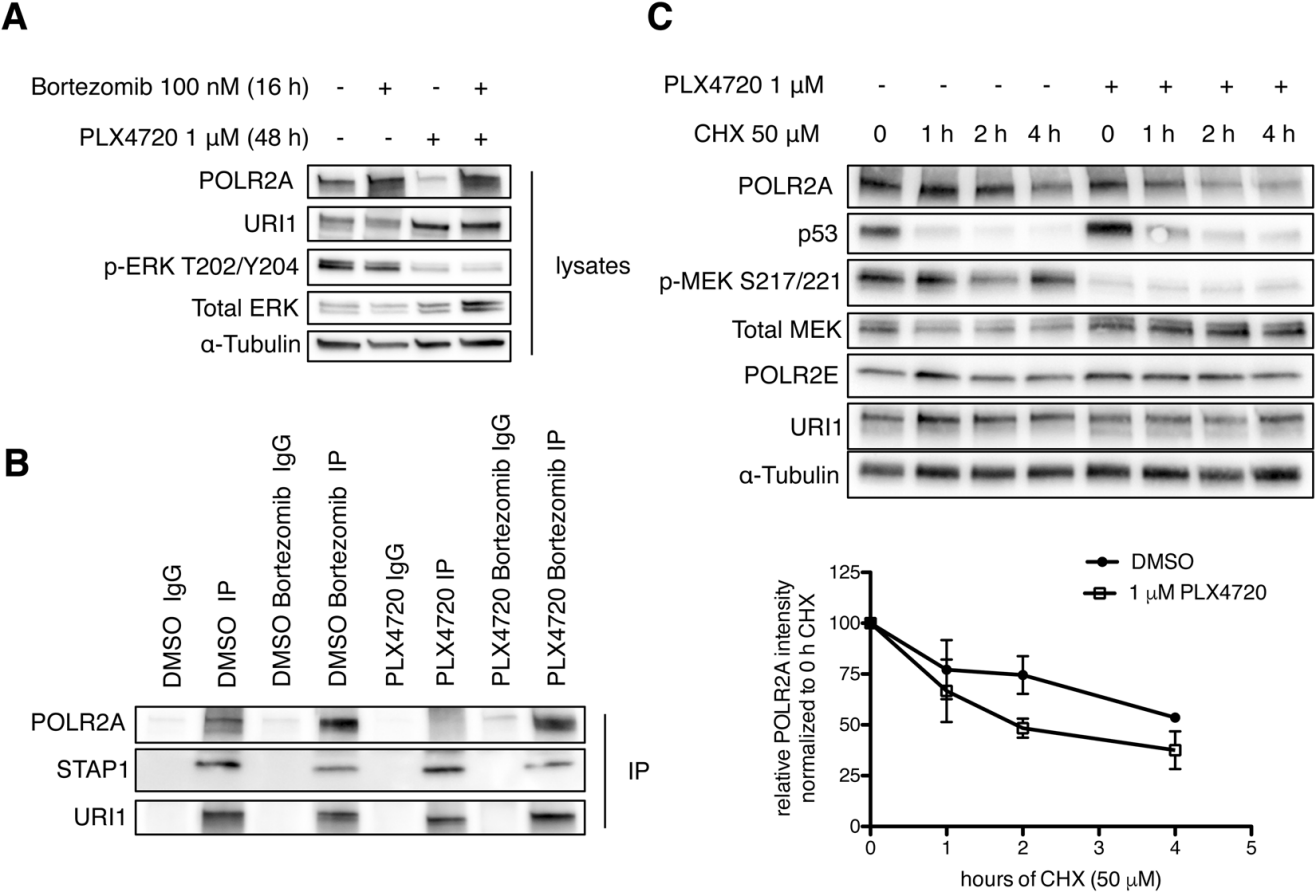
POLR2A is destabilized by inhibition of oncogenic BRAF^V600E^ signaling. (**A**) Representative immunoblot of A375-P treated with PLX4720 or DMSO for 48 hours. During the last 16 hours both PLX4720 and DMSO samples were treated with the proteasome inhibitor Bortezomib or vehicle control (n = 3). α-Tubulin served as a loading control. (**B**) URI1 co-immunoprecipitation of lysates described in (**A**) (n = 3). (**C**) Representative immunoblot of A375-P cells treated with PLX4720 or DMSO control for 16 hours, followed by cycoheximide treatment for the indicated timepoints. The lower panel shows the quantification of three independent biological replicates, normalized to the respective 0 CHX timepoint and α-Tubulin loading control (mean ± SD).

To study the reason for the observed POLR2A downregulation under BRAF^V600E^ inhibition, we inhibited proteasomal degradation with Bortezomib in A375-P cells treated with PLX4720 for 48 h. Bortezomib, added for 16 h, stabilized POLR2A both in DMSO- and PLX4720-treated cells to comparable levels (Fig. 2A). Furthermore, Bortezomib-stabilized POLR2A was able to bind to the URI1 chaperone (Fig. 2B). These data indicate that POLR2A binding to the URI1 complex mainly depends on POLR2A protein stability.

Blocking *de novo* protein synthesis with cycloheximide in A375-P cells demonstrated a rather short half-life of POLR2A. In cells treated with PLX4720 the protein half-life was even decreased (Fig. 2B). In contrast, the stability of URI1 and the URI1 interactor POLR2E was not affected by cycloheximide nor by PLX4720 treatment. The downregulation of p53 upon cycloheximide treatment was immediate and not affected by PLX4720. These data suggest that BRAF^V600E^ oncogenic signaling controls POLR2A protein stability and, by this, RNA polymerase II levels. Shutdown of this signaling pathway in melanoma cell lines leads to a decrease of POLR2A stability and therefore diminishes its protein levels over time.

### α-amanitin induces massive cell death of PLX4720-treated melanoma cell lines

**Figure 3:**
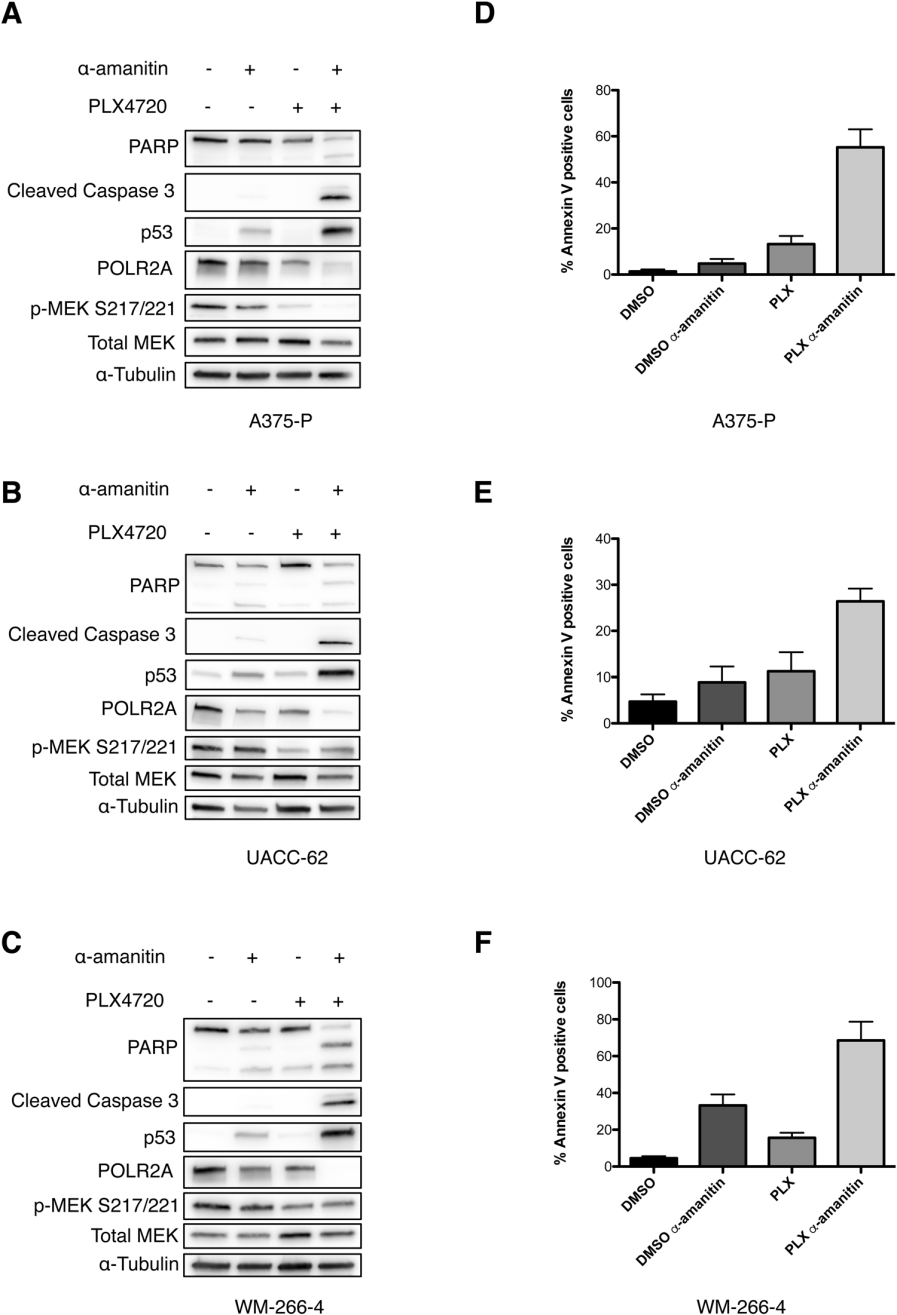
α-amanitin induces massive cell death in PLX4720-treated melanoma cell lines. (**A**–**C**) Representative immunoblots for POLR2A and apoptosis markers in indicated melanoma cell lines treated with α-amanitin (1 μg/ml in A375-P and 0.25 μg/ml in UACC-62 and WM-266-4) alone or in combination with 1 μM PLX4720 (n = 3). P-MEK, total MEK and α-Tubulin served as a treatment and loading control. (**D**–**F**) Apoptosis measurements of indicated melanoma cell lines by Annexin V-FITC and propidium iodide double staining after treatment with α-amanitin alone or in combination with PLX4720. Percentage of Annexin V positive cells for each condition is displayed. Data are mean ± SD (n = 3 biological replicates).

Since inhibition of oncogenic BRAF^V600E^ signaling in melanoma cells decreased POLR2A stability, we investigated whether the RNA polymerase II inhibitor α-amanitin might exhibit synergistic effects in combination with PLX4720. Therefore, we treated the three BRAF^V600E^-mutated melanoma cell lines A375-P, UACC-62 and WM-266-4 either with α-amanitin alone or in combination with PLX4720 for 72 hours and assessed POLR2A protein levels as well as PARP and Caspase-3 cleavage by western blot (Fig. 3A–C). Based on the data of Liu *et al*. in CRC cell lines^18^ with hemizygous *POLR2A* deletions we chose low concentrations of α-amanitin (0.25 μg/ml for UACC-62 and WM-266-4 and 1 μg/ml for the more resistant A375-P). Whereas α-amanitin alone had only modest effects on POLR2A levels and cleavage of apoptotic proteins, the combination with PLX4720 almost completely abolished POLR2A and caused a massive cleavage of PARP and Caspase-3 as well as an up-regulation of p53 (Fig. 3A–C). In addition, apoptosis was quantified by flow cytometry using Annexin V/propidium-iodide (PI) double staining (Fig. 3D–E and Supplementary Fig. 3A–C). The combination treatment significantly increased apoptotic cell death in all the three cell lines tested. In contrast, the combination of PLX4720 and α-amanitin did not increase the apoptotic rate in the BRAF- and NRAS-wildtype melanoma cell line MeWo (Fig. S3D,E).

### Inhibition of oncogenic activated MAPK signaling creates a collateral vulnerability towards α-amanitin similar as *POLR2A* hemizygous deletion

**Figure 4:**
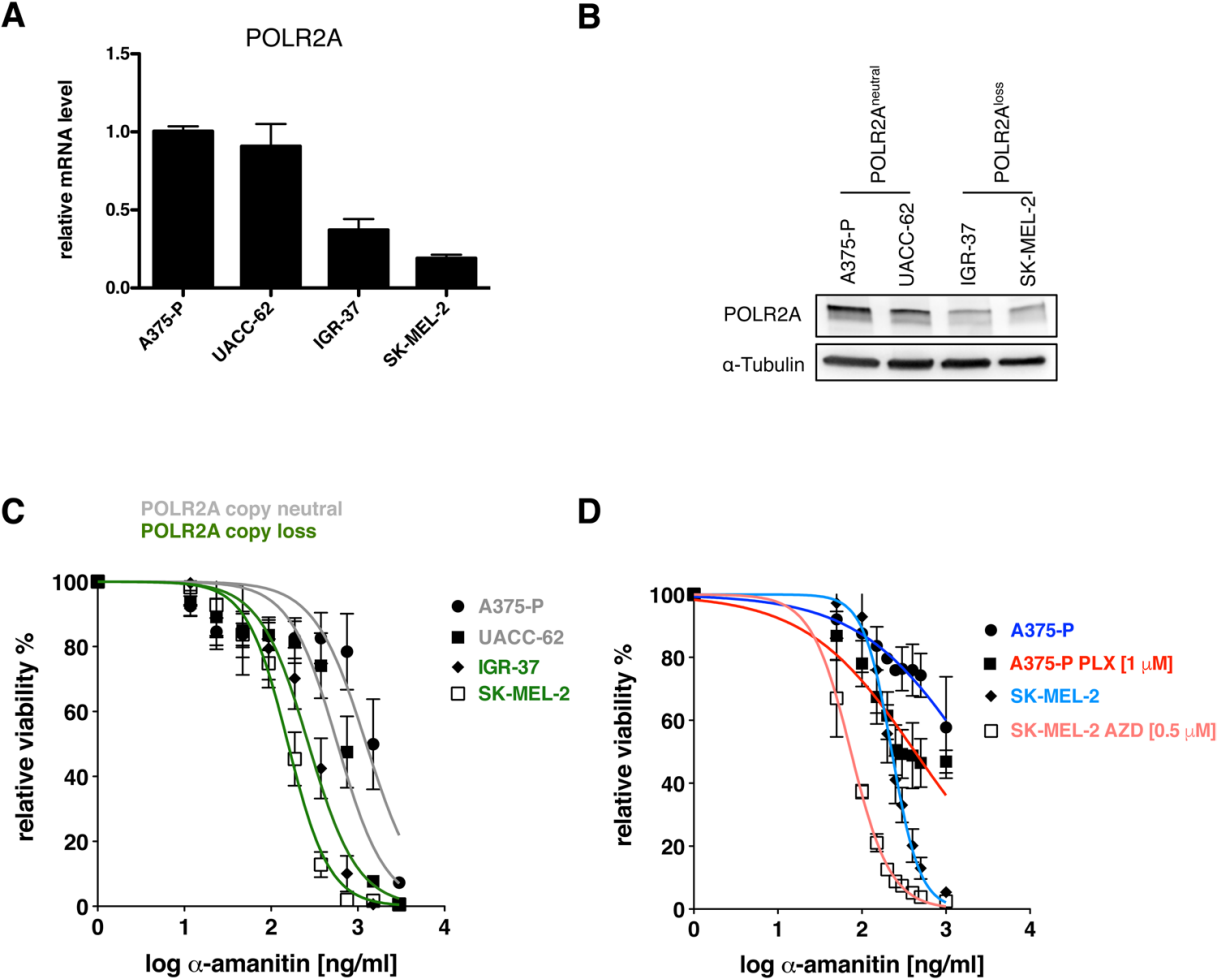
Inhibition of oncogenic activated MAPK signaling creates a collateral vulnerability towards α-amanitin, similar as *POLR2A* hemizygous deletion. (**A**) *POLR2A* expression in melanoma cell lines with two copies of *POLR2A* (A375-P, UACC-62) or a monoallelic loss of one copy (IGR-37, SK-MEL-2). Each value represents the amount of mRNA relative to that in A375-P cells, which was arbitrarily defined as 1. *TBP* was used as the invariant control. Data are mean ± SD (n = 3 biological replicates). (**B**) Representative immunoblot for POLR2A in melanoma cell lines used in (**A**) (n = 3). α-Tubulin served as a loading control. (**C**) Drug response curves of indicated melanoma cell lines treated with increasing concentrations of α-amanitin. Data are mean ± SD (n = 3 biological replicates). (**D**) Drug response curves to α-amanitin of A375-P and SK-MEL-2 pre-treated (for 16 h) either with the indicated MAP-Kinase inhibitor or DMSO as control. Data are mean ± SD (n = 3 biological replicates).

Liu *et al*. (2015) showed that CRC cell lines with only one allele of *POLR2A* and therefore lower POLR2A protein levels show an increased sensitivity to α-amanitin^18^. As *POLR2A* is a flanking gene of the tumor suppressor gene *TP53* on chromosome 17p, it is co-deleted in approximately half of CRC cases^18^. In melanoma *TP53* deletions are not as frequent as they are in CRC. However, hemizygous deletions of *TP53* and therefore *POLR2A* can be found in around one third of melanoma cases according to the TCGA database (http://portals.broadinstitute.org/tcga/home). Based on data from the Cancer Cell Line Encyclopedia (CCLE) database we chose two melanoma cell lines with a hemizygous deletion of *POLR2A* and two control cell lines with two *POLR2A* copies^25^. Similar as in CRC, melanoma cell lines with a hemizygous loss of *POLR2A* (*i.e*., IGR-37, SK-MEL-2) had significantly lower levels of *POLR2A* mRNA and protein than the *POLR2A* copy neutral cell lines A375-P and UACC-62 (Fig. 4A,B). To assess if the former are more sensitive to α-amanitin, we performed α-amanitin dose response curves. Indeed, both IGR-37 and SK-MEL-2 cells showed significantly lower IC50 values, 269.4 ng/ml (95% CI, 226.5 to 320.4 ng/ml) and 156.8 (95% CI, 141.1 to 174.1 ng/ml) respectively, compared to A375-P and UACC-62 cells with IC50 values of 1248 ng/ml (95% CI, 953.7 to 1632 ng/ml) and 581.7 ng/ml (95% CI, 478.0 to 707.9 ng/ml), respectively (Fig. 4C). Given the effect of BRAF^V600E^ and MEK1/2 inhibitors on POLR2A protein levels we examined whether the addition of a MAPK inhibitor would influence the IC50 values to α-amanitin independent of the *POLR2A* copy number status. Therefore, we treated the *POLR2A* loss and neutral cell lines SK-MEL-2 and A375-P, respectively, with a MAPK inhibitor followed by treatment with different α-amanitin concentrations. Indeed, treatment of SK-MEL-2 (POLR2A loss; NRAS^Q61R^) and A375-P (POLR2A neutral; BRAF^V600E^) with AZD6244 or PLX4720, respectively, increased the α-amanitin sensitivity significantly (IC50: 226.6 (95% CI, 210.3 to 244.1) vs. 73.88 (95% CI, 69.09 to 79.00) ng/ml and 1845 (95% CI, 1108 to 3070) vs. 427.2 (95% CI, 331.2 to 551.1) ng/ml respectively) (Fig. 4D).

## Discussion

In this study we describe a novel vulnerability of melanoma cell lines treated with MAPK inhibitors to the RNA polymerase II inhibitor α-amanitin. The underlying cause of this vulnerability is most likely a destabilization of the large RNA polymerase II subunit POLR2A by blocking oncogenic ERK signaling. Furthermore, our study sheds light onto how ERK signaling controls the assembly of RNA polymerase II holoenzymes.

In contrast to CDK9 (Cyclin-dependent kinase 9) inhibitors, α-amanitin not only transiently inhibits the transcription machinery but also leads to the degradation of POLR2A^20,21^. This might explain its synergistic effect on apoptosis induction observed in combination with MAPK inhibition in melanoma cells. Indeed, the combination of the CDK9 inhibitor Alvocidib with PLX4720 did not have the same effect as α-amanitin combined with PLX4720 (Supplementary Fig. 4). Since cell growth and proliferation increase the transcription rate of a cell^26^, it has to augment the availability of its transcription machinery. Here we present evidence that oncogenic activated ERK signaling does this at least in part by controlling the stability of the large RNA polymerase II subunit POLR2A.

Theoretically, the dependency of a BRAF^V600E^ inhibitor-treated quiescent cell on transcription should decrease. However, there might be a certain threshold of POLR2A protein levels necessary for cell survival and the combined effects of acute BRAF^V600E^ inhibition and α-amanitin treatment inducing POLR2A degradation could push cells towards apoptosis.

Due to its strong liver toxicity α-amanitin cannot be used *in vivo*, unless it is administered as an antibody-drug conjugate. Liu *et al*. conjugated α-amanitin to an anti-epithelial cell adhesion molecule (EpCAM) antibody and demonstrated complete tumor regression in mouse models of human colorectal cancer with a hemizygous loss of *POLR2A*^18^. The development of a melanoma-specific α-amanitin antibody-drug conjugate and its combination with BRAF^V600E^ inhibitors in preclinical melanoma models will be an important next step to further validate our findings. Antibodies targeting the transmembrane protein GPNMB (glycoprotein NMB) are the best characterized targets for antibody-drug conjugates in melanoma^27^ Indeed, for *in vivo* studies GPNMB antibodies coupled to α-amanitin might be an ideal candidate for a combination with a BRAF^V600E^ inhibitor, as GPNMB expression seems to slightly increase in PLX4720-treated cells (Supplementary Fig. 5). However, the safety of α-amanitin coupled to an antibody in humans is so far unknown because of a lack of clinical studies and has to be carefully addressed. Taken together, combining α-amanitin with BRAF^V600E^ inhibitors could potentially help to induce an apoptotic response in otherwise quiescent melanoma cells that represent a reservoir for the subsequent development of resistance to BRAF^V600E^ inhibition alone.

## Materials and Methods

### Cell lines and culture

Cells were cultivated at 37 °C in 21% O_2_ and 5% CO_2_ in a humidified incubator. The following melanoma cell lines and culture media were used: A375-P (R. Hynes, MIT), WM-266-4, A2058, SK-MEL-28, SK-MEL-2, MeWo and HEK-293T cells (ATCC) were cultured in Dulbecco’s modified Eagle’s medium (DMEM) supplemented with 10% FCS (Ambimed), 2 mM L-glutamine (Gibco) and 1% non-essential amino acids (NEAA; Gibco). UACC-62 cells (NCI-60) were cultured in RPMI-1640 medium supplemented with 10% FCS and GlutaMax (Gibco). IGR-37 cells (ATCC) were cultured in DMEM supplemented with 15% FCS.

All cell lines were regularly tested for mycoplasma contamination with a PCR-based assay (A8994; AppliChem). Cell line profiling was performed using highly-polymorphic short tandem repeat loci (STRs) and the DNA profile was compared with databases (Microsynth).

### Reagents

PLX4720 (S2807), AZD6244 (S1008), Bortezomib (S1013) and Alvocidib (S2679) were purchased from Selleck Chemicals and dissolved in DMSO (dimethyl sulfoxide) as 1 or 10 mM stock solutions and stored at −80 °C. Cycloheximide (Sigma-Aldrich) was dissolved in water as a 50 mM stock solution and stored at 4 °C. α-amanitin (A2263; Sigma-Aldrich) was dissolved in water as 1 mg/ml stock solution and stored at −20 °C.

### Cell lysis, immunoblotting and antibodies

Cells were washed twice with ice-cold phosphate-buffered saline (PBS), scraped from the dish, transferred to an Eppendorf tube and centrifuged at 2400 rpm in a tabletop centrifuge for 5 min; for apoptosis experiments cell culture supernatants were collected and combined with cells which were harvested by trypsinization. Cell pellets were lysed in TNN buffer (50 mM Tris, pH 7.5; 250 mM NaCl; 5 mM EDTA; 0.5% NP-40; 50 mM NaF; 0.5 mM EGTA) containing protease and phosphatase inhibitors (cOmplete and PhosSTOP, respectively; Roche) for 20 min on ice. The samples were centrifuged at full-speed in a tabletop centrifuge for 10 min and protein concentrations were determined using the Bradford assay (Bio-Rad). Protein concentrations were adjusted using TNN and 5x Laemmli buffer and boiled at 95 °C for 5 min. Equal amounts of proteins were separated by SDS-PAGE (sodium dodecyl sulfate polyacrylamide gel electrophoresis) using 4–20% Mini-PROTEAN TGX Stain-Free Gels (Bio-Rad) and transferred to Trans-Blot Turbo PVDF Transfer Packs (Bio-Rad) using the Trans-Blot® TurboTM system (Bio-Rad). Membranes were blocked with 5% milk in Tris-buffered saline containing 0.05% Tween 20 (TBST) and incubated with the primary antibody over-night at 4 °C. After washing with TBST, incubation with the corresponding horseradish peroxidase-conjugated secondary antibodies (Invitrogen®) was performed for 1 h at room temperature, membranes were washed again in TBST, developed using a homemade ECL solution and visualized on the ImageQuant LAS4000® mini (GE Healthcare).

The URI1 (mAb 58.1) and STAP1/UXT (mAb 105.128) monoclonal antibodies have been described previously and were used in a 1:100 dilution^14^. p44/42 MAPK (Erk1/2) (#9102), Phospho-p44/42 MAPK (Erk1/2) (Thr202/Tyr204) (#9101), MEK1/2 (#9122), Phospho-MEK1/2 (Ser217/221) (#9121) and Cleaved Caspase-3 (#9664) antibodies were purchased from Cell Signaling Technology and used in a 1:1000 dilution. Alpha-tubulin (ab18251) (1:2000), POLR1A (ab139130) (1:500) and POLR3A (ab96328) (1:500) were purchased from Abcam, PARP (556362) (1:1000) and p53 (554293) (1:1000) from BD Pharmingen, POLR2A (39097) (1:1000) from Active Motif, and POLR2E (3B5) (NBP2-00482) (1:1000) from Novus Biologicals.

### RNA extraction, cDNA preparation and quantitative real-time RT-PCR (qPCR)

Total RNA was isolated from cultured cells using the NucleoSpin RNA II kit (Macherey-Nagel) according to the manufacturer’s instructions. RNA concentration was determined using a NanoDrop 2000 (Thermo Scientific). 1 or 2 μg of total RNA were reverse transcribed using the High-Capacity RNA-to-cDNA™ Kit (Cat. No. 4368813; Applied Biosystems) according to the manufacturer’s instruction and the cDNA was diluted to 5 ng/μl. For qPCR a 20 μl reaction mix was prepared in a Roche LightCycler 480 Multiwell Plate (1 μl 5 μM forward primer, 1 μl 5 μM reverse primer, 10 μl KAPA SYBR® FAST, 3 μl water and 5 μl cDNA) and run on a Roche LightCycler LC480, using the following program: 5 min pre-incubation at 95 °C, 45 amplification cycles (10 sec. 95 °C, 10 sec. 60 °C, 10 sec. 72 °C). A melt curve analysis was carried out to confirm the specific amplification of a target gene and absence of primer dimers. Relative mRNA amount was calculated using the comparative threshold cycle (CT) method. *TBP* (TATA-box binding protein) was used as the invariant control. The following primer pairs were used: *TBP* (forward: 5′-TTCGGAGAGTTCTGGGATTGT-3′; reverse: 5′-TGGACTGTTCTTCACTCTTGG-3′); *SPRY2* (forward: 5′-ATCAGATCAGAGCCATCCGAA-3′; reverse: 5′-TGGAGTCTCTCGTGTTTGTGC-3′); *CCND1* (forward: 5′-GAACAAACAGATCATCCGCAAAC-3′; reverse: 5′-GCGGTAGTAGGACAGGAAGTTG-3′); *POLR2A* (forward: 5′-CATCAAGAGAGTCCAGTTCGG-3′; reverse: 5′-CCCTCAGTCGTCTCTGGGTA-3′).

### URI1 co-immunoprecipitation

Cell lysis was performed as described above. Lysates were precleared with 100 μl of recombinant Protein G Agarose (ThermoFisher Scientific) for 30 min on a slowly rotating wheel at 4 °C. After preclearing 900 μg of total protein was mixed with 2 μg of the anti-URI1 monoclonal antibody (clone 179-2-1)^14^ or mouse IgG control, the volume adjusted to 500 μl with TNN and incubated for 2 h on a slowly rotating wheel at 4 °C. After adding 100 μl of Protein G Agarose the samples were incubated for another hour and subsequently washed four times with 900 μl TNN buffer. Finally, 10 μl of 5x Laemmli buffer was added to the Agarose, samples were vortexed for 15 sec and boiled at 95 °C for 5 min.

### Apoptosis assays by Annexin V and Propidium iodide staining

Cells treated with α-amanitin for 72 hours and the control cells were harvested by trypsinization collecting all supernatants, washed twice with ice-cold PBS and incubated with Annexin V-FITC conjugate (A13199; ThermoFisher Scientific)-containing Annexin V binding buffer (10 mM Hepes/NaOH, pH 7.4; 140 mM NaCl; 2.5 mM CaCl_2_) for 15 min at room temperature. Propidium Iodide (PI) was added just prior to analysis by flow cytometry. Stained cells were analyzed using a BD Accuri™ C6 counting 10’000 events per sample. Unstained and single stained controls were included for color compensation. The data were analyzed using FlowJo software (Tree Star, Inc.).

### Dose-response curves

For dose-response curves 1’500 cells or 4’500 cells (PLX4720 and AZD6244 treated plates) were seeded in a 96-well plate. On the following day nine different α-amanitin or Alvocidib concentrations plus treatment control were added and incubated for 72 hours. After the incubation period cells were washed four times with PBS using a HydroSpeed™ plate washer (TECAN) and incubated for 30 min at 37 °C and 5% CO_2_ with PrestoBlue® Cell Viability Reagent (A13262; ThermoFisher Scientific) diluted 1:10 in PBS. The converted fluorescent dye was measured using the Infinite® M1000 PRO microplate reader (TECAN). Values were normalized to solvent control.

### URI1 mass spectrometry

URI1 co-immunoprecipitation was performed as described with URI1 antibody coupled to recombinant Protein G Agarose. 5 mg of lysate were used for URI1 IP/IgG control of PLX4720 and DMSO control samples. Samples were incubated for 4 hours in 15 ml Falcon tubes on a slowly rotating wheel followed by washing of the Agarose. The resuspended Agarose was loaded on Micro Bio-Spin® columns (Bio-Rad) and washed four times with 1 ml TNN buffer followed by another four times with 1.5 ml of a detergent free TNN buffer until bubbles were completely removed. Elution was performed with 150 μl 1 M glycine (pH 2.5). Immediately after elution, the eluates were neutralized with 5 μl 1 M NH_4_HCO_3_ (pH 8.8) and pH was verified with pH-indicator strip (Merck). 200 mM Tris(2-carboxyethyl)phosphine (TCEP) in NH_4_CO_3_ was added to the eluates and samples were incubated at 37 °C for 40 min, followed by 200 mM iodoacetamide to a final concentration of 10 mM and incubation at room temperature for 30 min in the dark. For peptide digestion 1 μg of trypsin (Sequencing Grade Modified Trypsin, Promega) was added to the eluates and incubated at 37 °C overnight. The next day, acidification of the samples with 10% formic acid was performed to reach a pH of 2–3 as tested with pH-indicator strips. 50% acetonitrile (ACN) was added to a final concentration of 1%. Next, samples were loaded on prepared Ultra-Micro SpinColumnsTM (Harward), washed three times with 200 μl 0.1% formic acid/5% ACN and peptides were eluted with 100 μl 0.1% formic acid/50% ACN. Samples were immediately processed to MS. LC-MS/MS was performed as previously described^28^.

Progenesis software (version 4.1; Nonlinear Dynamics, Newcastle, UK) was used for intensity-based label-free quantification. After selecting one sample as a reference, the retention times of all eluting precursor m/z values in all other samples within the experiment were aligned creating a list of “features” representing the same peptide in each sample. Features with two to six charges were included for further analysis. After alignment and feature filtering, replicate samples were grouped together, and raw abundances of all features were normalized. Briefly, for each sample, one unique factor is calculated and used to correct all features in the sample for experimental variation. Filtered peptide–spectrum matches (1% peptide–spectrum match FDR) were imported into Progenesis and matched to the respective features.

### Data presentation and analysis

Prism 6 software (GraphPad Software Inc.) was used for all statistical analyses and data presentations.

## Supplementary information

Supplementary Information
