# Supplementary material for: BRAF inhibition sensitizes melanoma cells to α-amanitin via decreased RNA polymerase II assembly
Source: Sci Rep. 2019 May 23;9:7779. doi: 10.1038/s41598-019-44112-7 (PMC6533289; doi:10.1038/s41598-019-44112-7)
Supplement: Supplementary file 1 — Supplementary Information [file 41598_2019_44112_MOESM1_ESM.pdf]

Supplementary Information

BRAF inhibition sensitizes melanoma cells to α-amanitin via decreased RNA polymerase II assembly

Lukas Frischknecht[#1], Christian Britschgi[#1,3], Patricia Galliker[1], Yann Christinat[1], Anton Vichalkovski[2], Matthias Gstaiger[2], Werner J. Kovacs[*1] and Wilhelm Krek[†1]

[1]Institute of Molecular Health Sciences, ETH Zurich, 8093 Zurich, Switzerland

[2]Institute of Molecular Systems Biology, ETH Zurich, 8093 Zurich, Switzerland.

[3]Current affiliation: Department of Medical Oncology and Hematology, University Hospital of Zurich and University of Zurich, 8091 Zurich, Switzerland.

[#]These authors contributed equally to this work.

[†]Deceased 29. August 2018

*Correspondence to: Werner J. Kovacs

Email: werner.kovacs@biol.ethz.ch

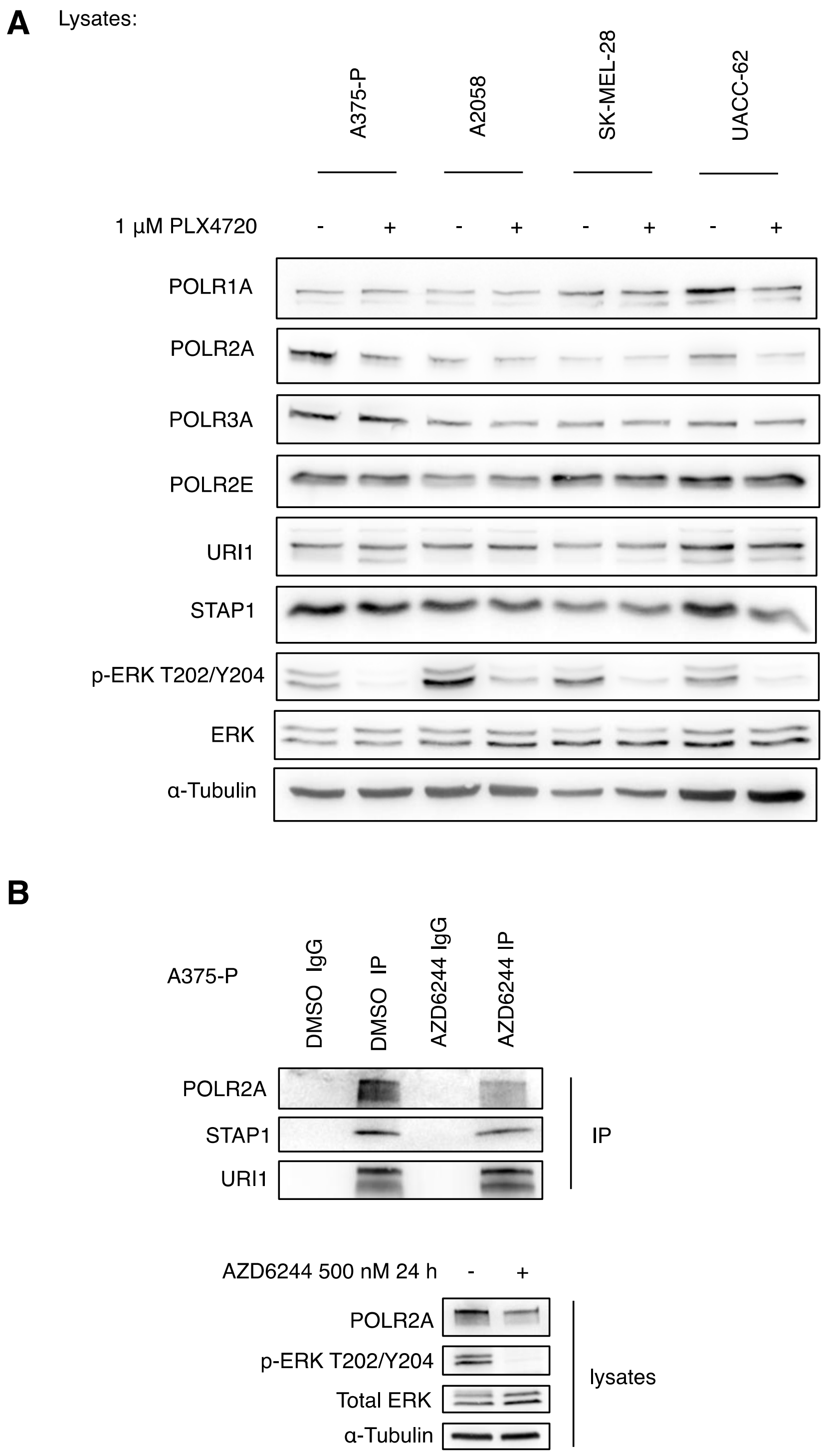

Figure S1. (A) Representative immunoblot of input lysates of the URI1 co-immunoprecipitation experiment shown in Figure 1. P-ERK, total ERK and α-Tubulin served as a treatment and loading control, respectively. (B) Representative URI1 co-immunoprecipitation of A375-P cells treated with 500 nM of the MEK1/2 inhibitor AZD6244. The lower panel shows the respective input lysates (n = 3).

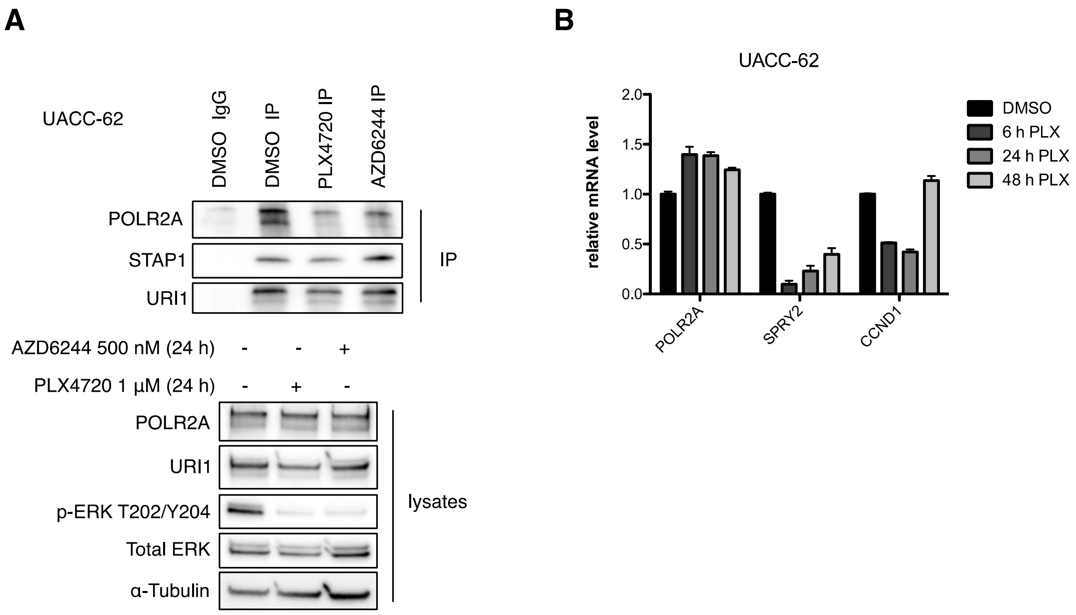

Figure S2. (A) Representative URI1 co-immunoprecipitation of UACC-62 cells treated with either 1 µM PLX4720, 500 nM AZD6244 or DMSO as control. The lower panel shows the respective input lysates (n = 3). (B) Expression of *POLR2A* and the ERK target genes *SPRY2* and *CCND1* after indicated time of PLX4720 treatment in UACC-62 cells. Each value represents the amount of mRNA relative to that in DMSO-treated cells, which was arbitrarily defined as 1. Data are mean ± SD (n = 3 biological replicates).

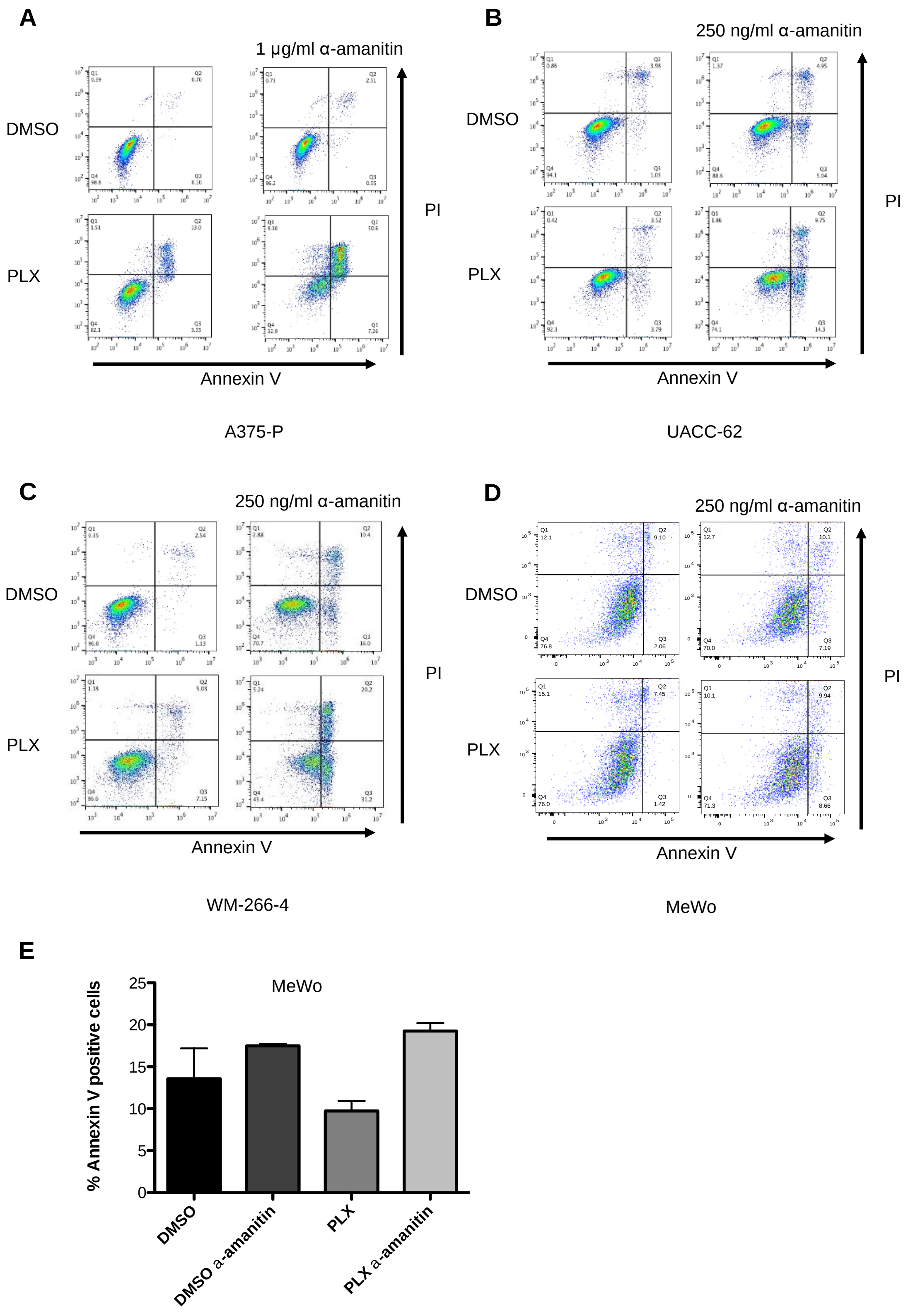

Figure S3. (A, B, C and D) Representative dot plot analysis of apoptosis measurement by Annexin V-FITC and propidium iodide double staining of indicated melanoma cell lines treated with the indicated α-amanitin concentration alone or in combination with 1 µM PLX4720 for 72 h. For the $BRAF^{V600E}$-mutated cell lines A375-P, UACC-62 and WM-266-4 the quantification of three independent experiments is shown in Figure 3. (E)

Quantification of three independent experiments for the BRAF and NRAS wild-type cell line MeWo.

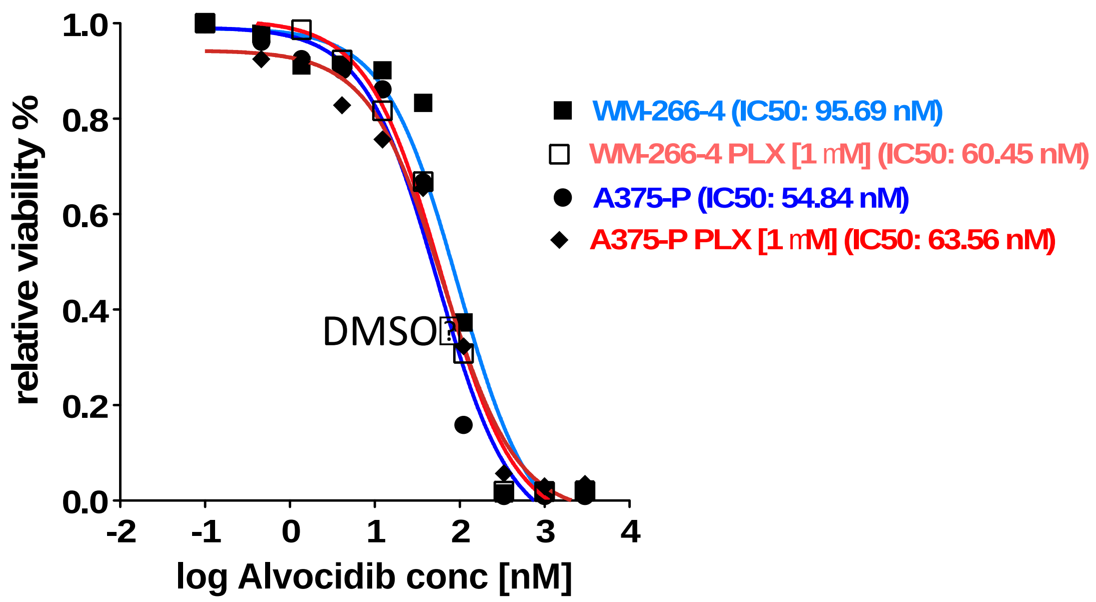

Figure S4. Drug response curves to Alvocidib of WM-266-4 and A375-P cells pre-treated (for 16 h) either with 1 μM PLX4720 or DMSO as control.

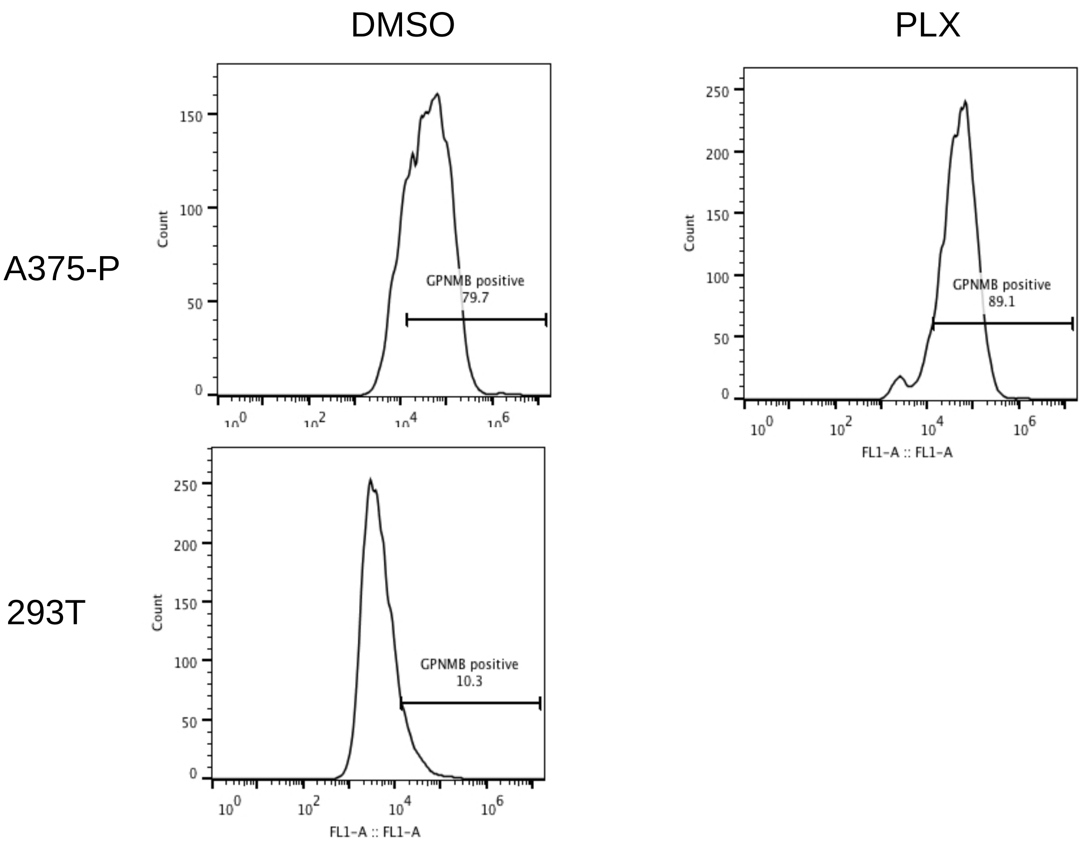

Figure S5. Flow cytometry analysis of A375-P cells treated for 24 h with 1 µM PLX4720 or DMSO as control and HEK 293T cells as a non-melanoma control cell line stained with an antibody against the transmembrane protein GPNMB (glycoprotein NMB).

Supplementary Table S1

UniProtKB name	UniProt	A375-P IgG DMSO	A375-P URI1 DMSO	A375-P IgG PLX	A375-P URI1 PLX
CUX1_HUMAN	P39880	0	147	0	122
CTR9_HUMAN	Q6PD62	0	90	0	72
RPAP3_HUMAN	Q9H6T3	0	81	0	74
RPC1_HUMAN	O14802	0	70	0	52
CDC73_HUMAN	Q6P1J9	0	66	0	56
SYEP_HUMAN	P07814	6	64	8	57
CASP_HUMAN	Q13948	0	62	0	51
AL1A3_HUMAN	P47895	8	61	9	72
STML2_HUMAN	Q9UJZ1	3	60	1	39
RMP_HUMAN	O94763	0	51	0	53
TFAP4_HUMAN	Q01664	0	51	0	25
RUVB2_HUMAN	Q9Y230	4	49	4	48
RUVB1_HUMAN	Q9Y265	7	44	8	50
WDR92_HUMAN	Q96MX6	0	39	0	37
PAF1_HUMAN	Q8N7H5	0	39	0	32
SYQ_HUMAN	P47897	1	37	4	30
WDR61_HUMAN	Q9GZS3	0	37	0	32
SYRC_HUMAN	P54136	5	36	5	36
ERCC5_HUMAN	P28715	0	34	0	24
PIHD1_HUMAN	Q9NWS0	0	29	0	28
SYIC_HUMAN	P41252	0	27	0	30
SYDC_HUMAN	P14868	0	24	0	20
SYMC_HUMAN	P56192	0	22	0	16
RPC2_HUMAN	Q9NW08	0	22	0	12

RPB1_HUMAN	P24928	0	21	0	8
LEO1_HUMAN	Q8WVC0	0	20	0	19
SNX30_HUMAN	Q5VWJ9	0	19	0	16
ATX2L_HUMAN	Q8WWM7	0	19	0	11
GBF1_HUMAN	Q92538	0	18	0	22
YLPM1_HUMAN	P49750	0	18	0	16
RPC5_HUMAN	Q9NVU0	0	18	0	11
PFD2_HUMAN	Q9UHV9	1	17	1	15
ZMYM1_HUMAN	Q5SVZ6	0	16	0	5
PABP1_HUMAN	P11940	4	16	5	10
YM012_HUMAN	Q9UF83	0	15	0	9
SYNE1_HUMAN	Q8NF91	0	15	0	8
HNRPC_HUMAN	P07910	4	14	0	13
PFD6_HUMAN	O15212	0	13	0	13
BACH1_HUMAN	O14867	0	13	0	2
DDX3X_HUMAN	O00571	2	13	3	4
ADDA_HUMAN	P35611	0	13	0	16
ACTBL_HUMAN	Q562R1	0	13	0	0
UXT_HUMAN	Q9UBK9	0	12	0	13
RPAB1_HUMAN	P19388	0	12	0	13
AIMP1_HUMAN	Q12904	0	12	0	9
PABP4_HUMAN	Q13310	3	12	0	6
SYK_HUMAN	Q15046	2	11	2	11
ARFG2_HUMAN	Q8N6H7	0	11	0	10
RPAC1_HUMAN	O15160	0	11	0	4
PZRN3_HUMAN	Q9UPQ7	0	9	0	14
PUR8_HUMAN	P30566	0	9	0	9
PDRG1_HUMAN	Q9NUG6	0	9	0	10

PP1A_HUMAN	P62136	0	9	0	9
RPC4_HUMAN	P05423	0	9	0	4
HMBX1_HUMAN	Q6NT76	0	8	0	5
PP1B_HUMAN	P62140	5	8	0	8
NEK2_HUMAN	P51955	0	8	0	0
ZBT10_HUMAN	Q96DT7	0	8	0	0
RPAB3_HUMAN	P52434	0	7	0	6
NUFP2_HUMAN	Q7Z417	0	7	0	2
CCD14_HUMAN	Q49A88	0	7	0	5
CDR2_HUMAN	Q01850	0	7	0	3
MYH10_HUMAN	P35580	0	6	0	6
AIMP2_HUMAN	Q13155	0	6	0	8
STX2_HUMAN	P32856	0	6	0	3
TR150_HUMAN	Q9Y2W1	0	6	3	6
SNX4_HUMAN	O95219	0	5	0	1
MCA3_HUMAN	O43324	0	5	0	5
CNST_HUMAN	Q6PJW8	0	5	0	5
ELOB_HUMAN	Q15370	2	5	2	2
TITIN_HUMAN	Q8WZ42	0	5	0	0
GCR_HUMAN	P04150	0	5	0	6
ADDG_HUMAN	Q9UEY8	0	5	0	10
BCLF1_HUMAN	Q9NYF8	0	5	0	1
MARK3_HUMAN	P27448	0	4	0	2
RPC8_HUMAN	Q9Y535	0	4	0	4
KTN1_HUMAN	Q86UP2	0	4	0	0
RPB2_HUMAN	P30876	0	4	0	0
LSM12_HUMAN	Q3MHD2	0	3	0	3
EIF3B_HUMAN	P55884	0	3	0	3

PIMT_HUMAN	P22061	0	3	0	3
RPAC2_HUMAN	Q9Y2S0	0	3	0	3
SUN2_HUMAN	Q9UH99	0	3	0	3
RPC10_HUMAN	Q9Y2Y1	0	3	0	2
FL2D_HUMAN	Q15007	0	3	0	0
RU2A_HUMAN	P09661	0	2	0	1
SYLC_HUMAN	Q9P2J5	0	2	0	3
PKD2_HUMAN	Q13563	0	2	0	2
ML12B_HUMAN	O14950	0	2	0	1
FMNL3_HUMAN	Q8IVF7	0	2	0	3
RPAP2_HUMAN	Q8IXW5	0	2	0	0
OSBP1_HUMAN	P22059	0	2	0	2
ZN609_HUMAN	O15014	0	2	0	1
AP2A2_HUMAN	O94973	0	2	0	0
IF4G2_HUMAN	P78344	0	2	0	0
CDR2L_HUMAN	Q86X02	0	2	0	0
ACBD5_HUMAN	Q5T8D3	0	2	0	0
RAB13_HUMAN	P51153	0	2	0	0
ITB4_HUMAN	P16144	0	2	0	0
RPB3_HUMAN	P19387	0	2	0	0
HERC1_HUMAN	Q15751	0	2	0	0
RN213_HUMAN	Q63HN8	0	2	0	0
VIR_HUMAN	Q69YN4	0	2	0	0
DB119_HUMAN	Q8N690	0	2	0	0
GPN3_HUMAN	Q9UHW5	0	2	0	0
LRRF1_HUMAN	Q32MZ4	0	1	0	1
PGAM5_HUMAN	Q96HS1	1	1	0	1
ATP5L_HUMAN	O75964	0	1	0	0

F195B_HUMAN	C9JLW8	0	1	0	2
DLG5_HUMAN	Q8TDM6	0	1	0	1
RUXGL_HUMAN	A8MWD9	0	1	1	1
EP15R_HUMAN	Q9UBC2	0	1	0	1
RBM14_HUMAN	Q96PK6	0	1	0	0
FIP1_HUMAN	Q6UN15	0	1	0	0
GBB3_HUMAN	P16520	0	1	0	0
SPY4_HUMAN	Q9C004	0	1	0	0
RPA1_HUMAN	O95602	0	0	0	8
PP1G_HUMAN	P36873	5	0	2	9
LRRN2_HUMAN	O75325	0	0	0	1
MTA2_HUMAN	O94776	0	0	0	2

Legend to Supplementary Table S1. Spectral counts of URI1 mass-spectrometry in A375-P cells treated with 1 μM PLX4720 for 16 h or DMSO as control.

Figure 1B

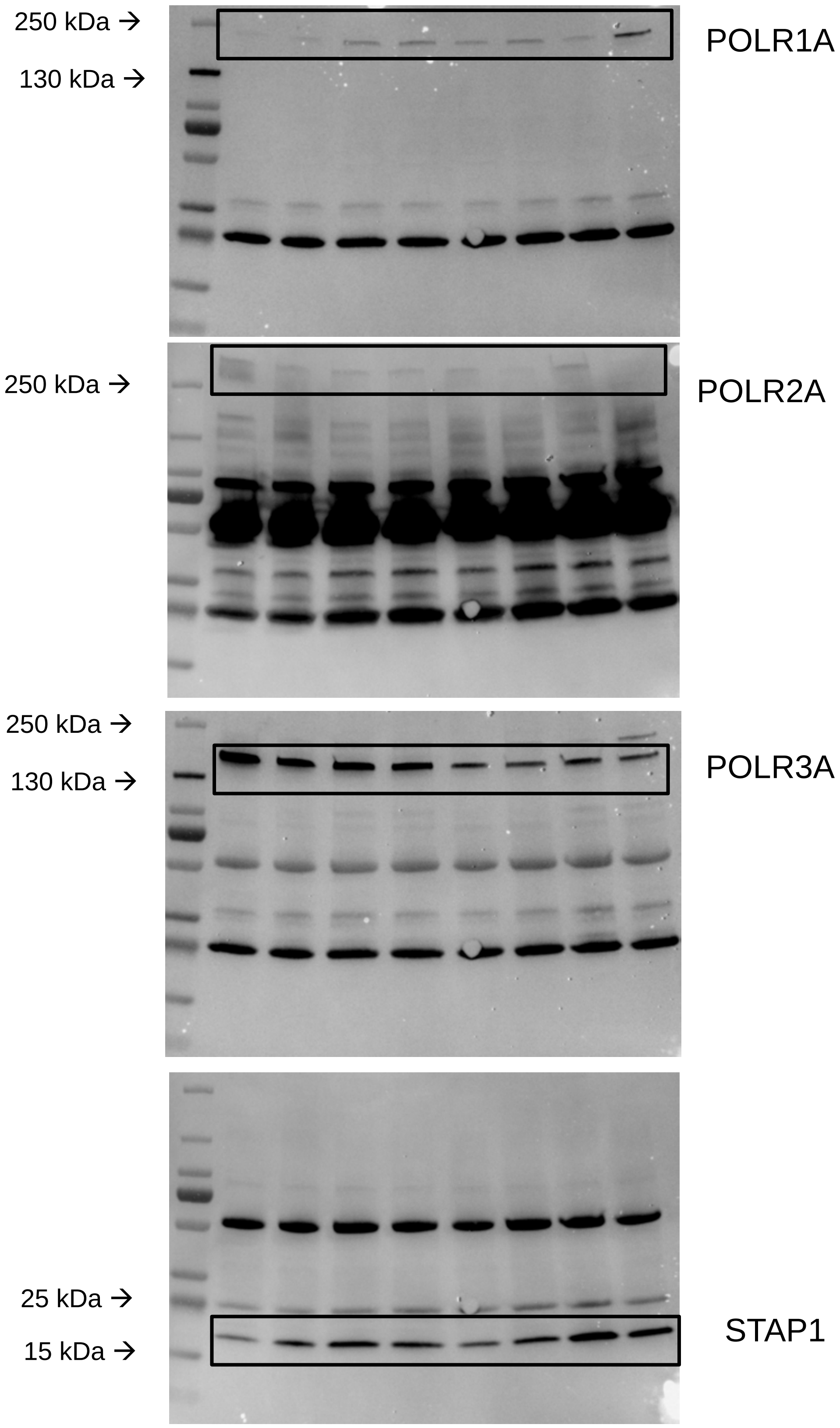
250 kDa →
130 kDa →
POLR1A
250 kDa →
POLR2A
250 kDa →
130 kDa →
POLR3A
25 kDa →
15 kDa →
STAP1

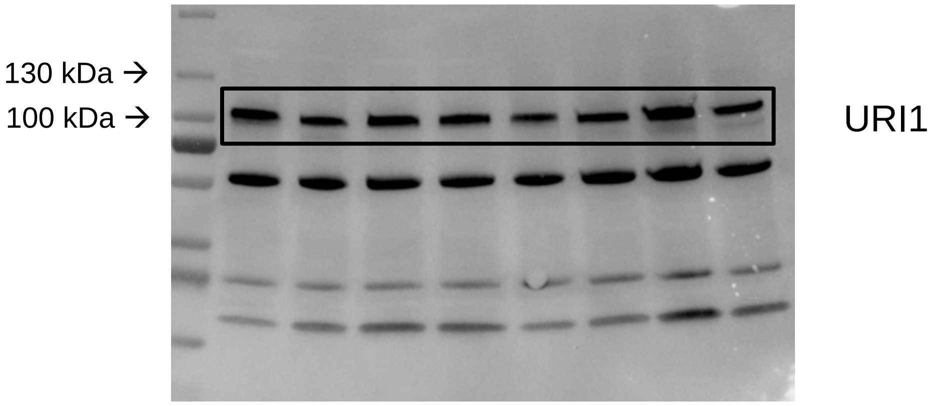
130 kDa →
100 kDa →
URI1

Figure 1C

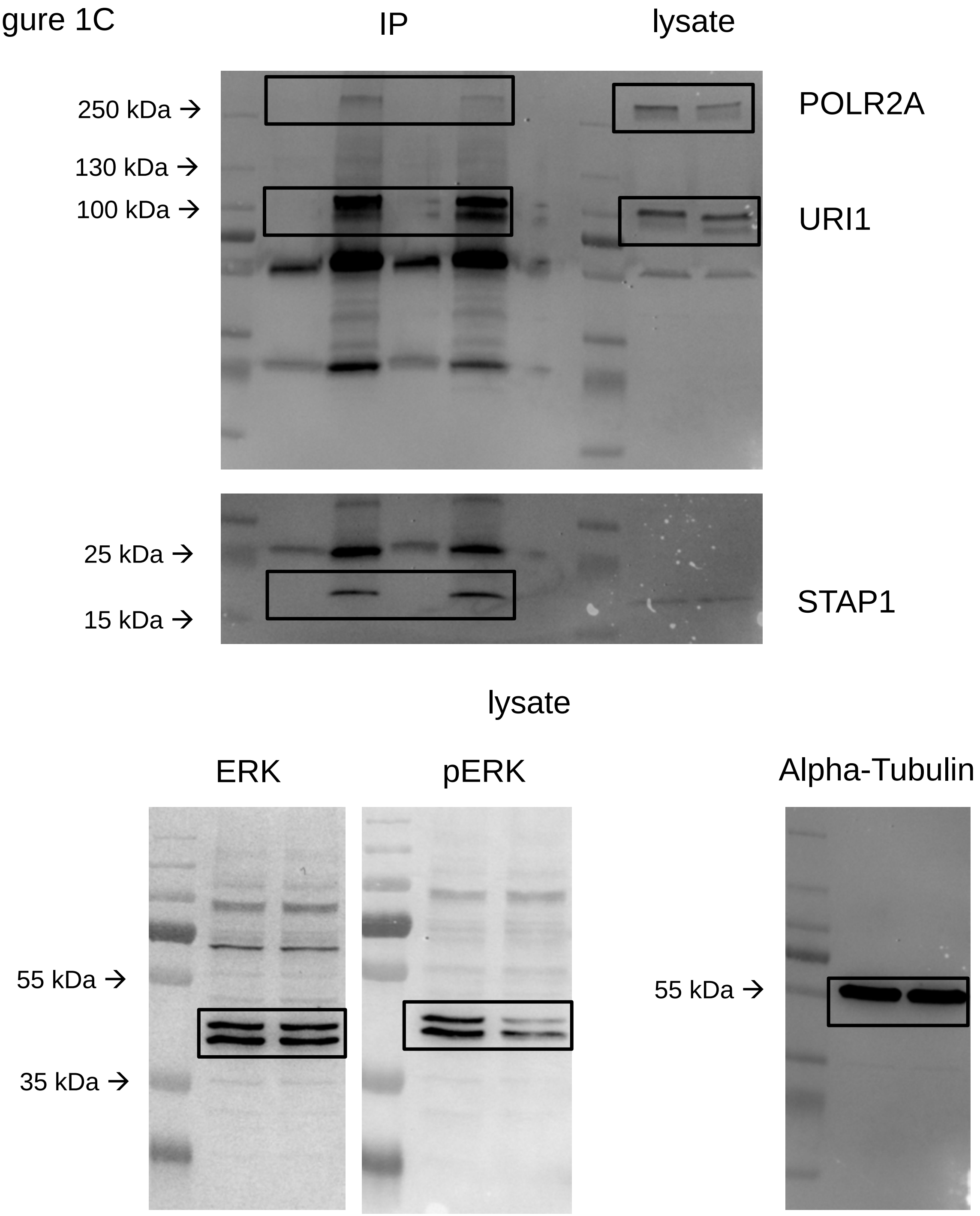
IP
lysate
250 kDa →
130 kDa →
100 kDa →
POLR2A
URI1
25 kDa →
15 kDa →
STAP1
lysate
ERK
pERK
Alpha-Tubulin
55 kDa →
35 kDa →
55 kDa →

Figure 1D

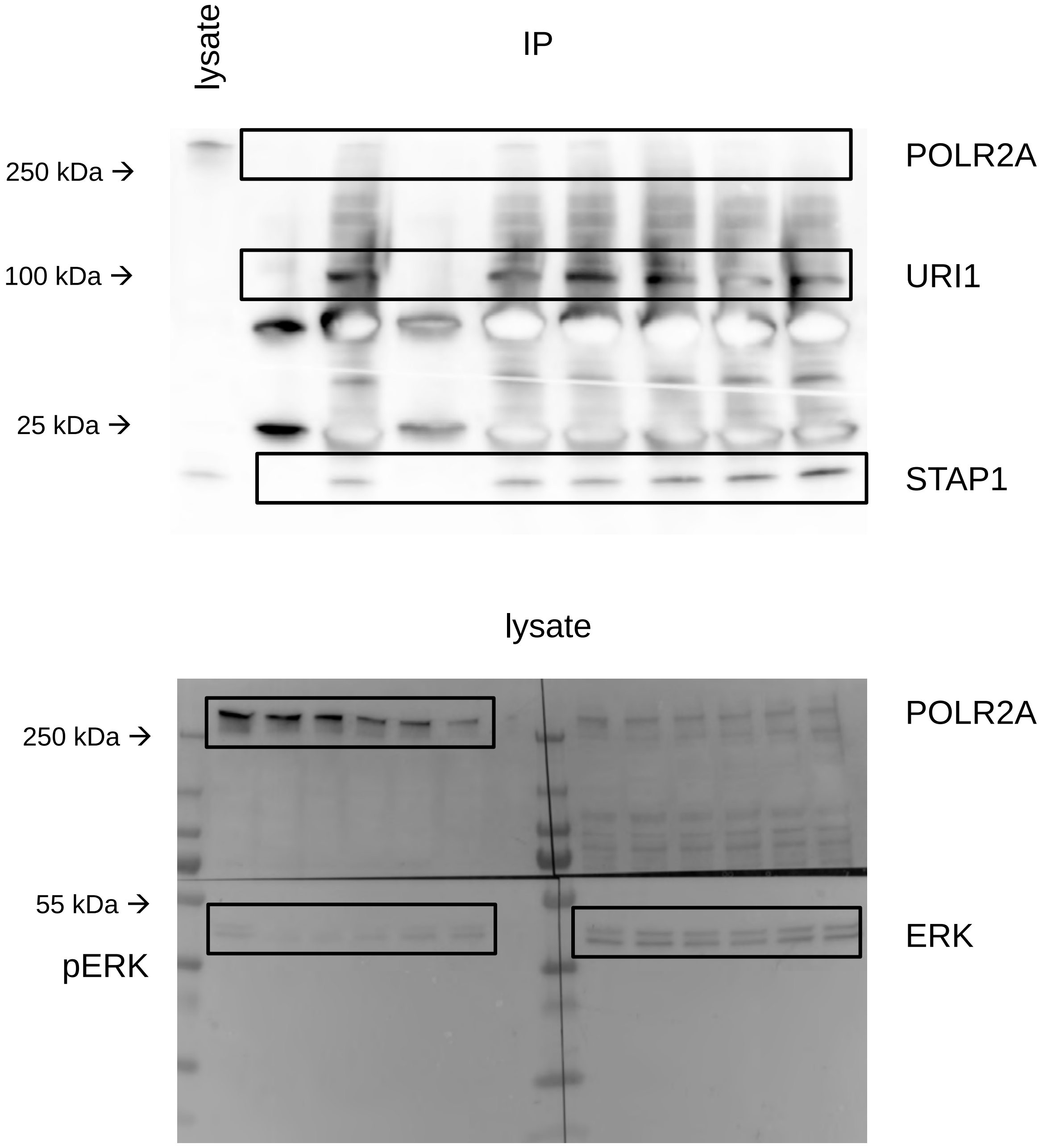
lysate
IP
250 kDa →
100 kDa →
25 kDa →
POLR2A
URI1
STAP1
lysate
250 kDa →
55 kDa →
pERK
POLR2A
ERK

Figure 2A

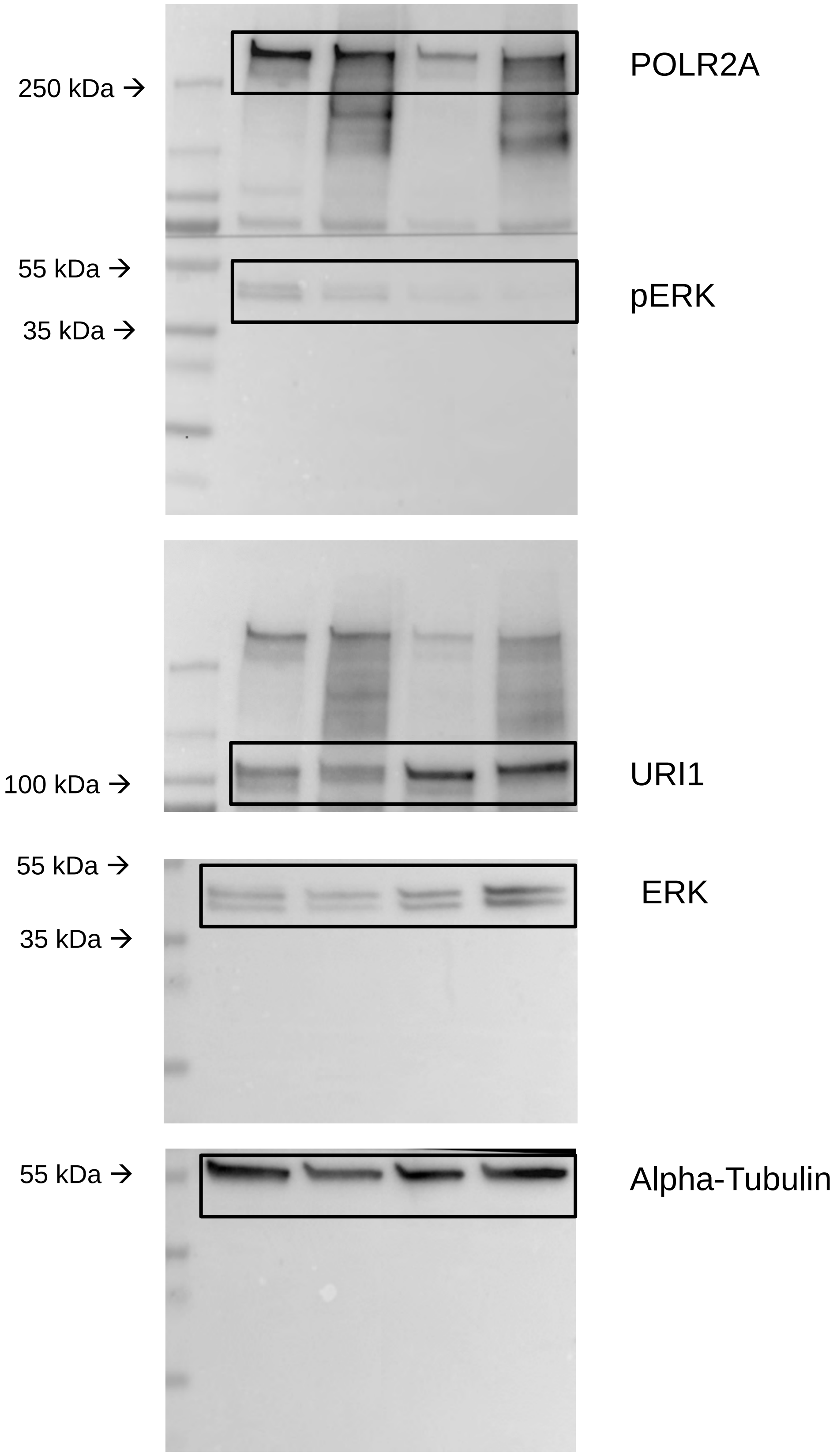
250 kDa →
POLR2A
55 kDa →
pERK
35 kDa →
100 kDa →
URI1
55 kDa →
ERK
35 kDa →
55 kDa →
Alpha-Tubulin

Figure 2B

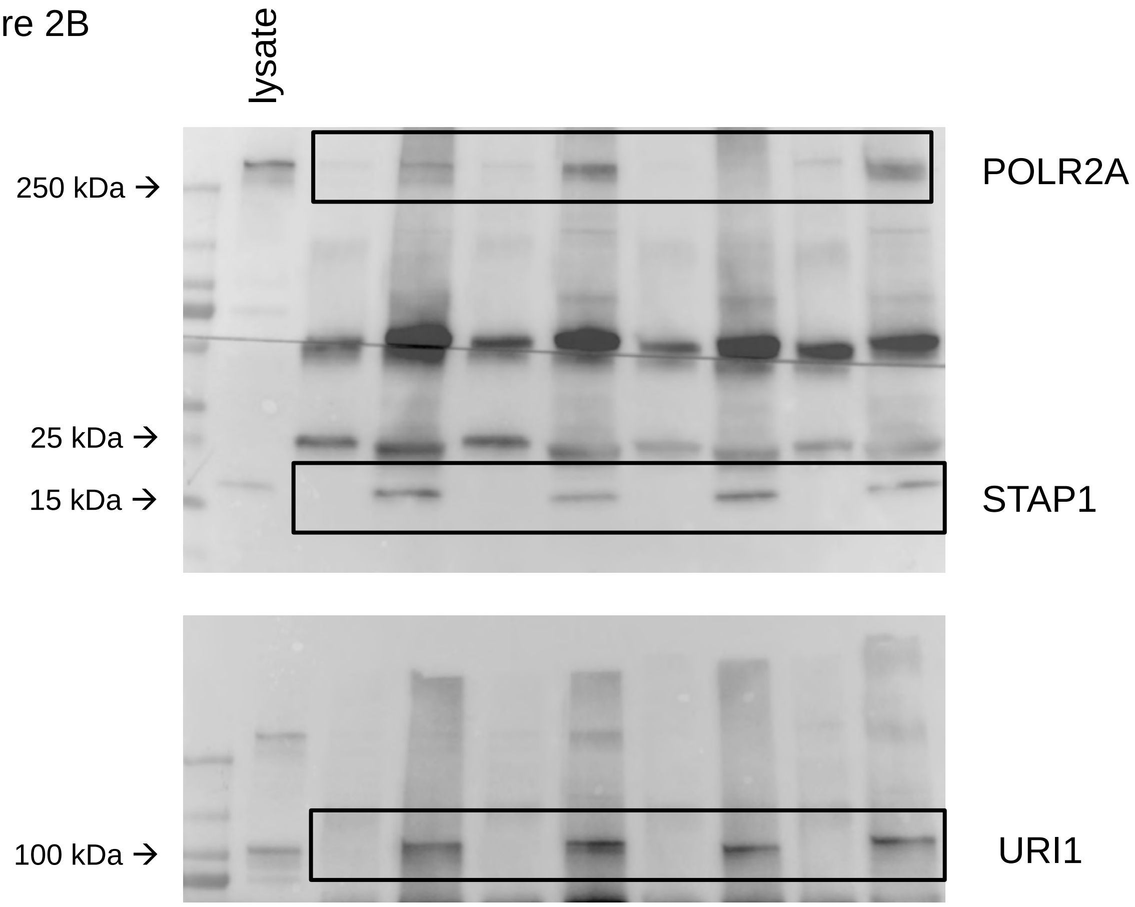

Figure 2C

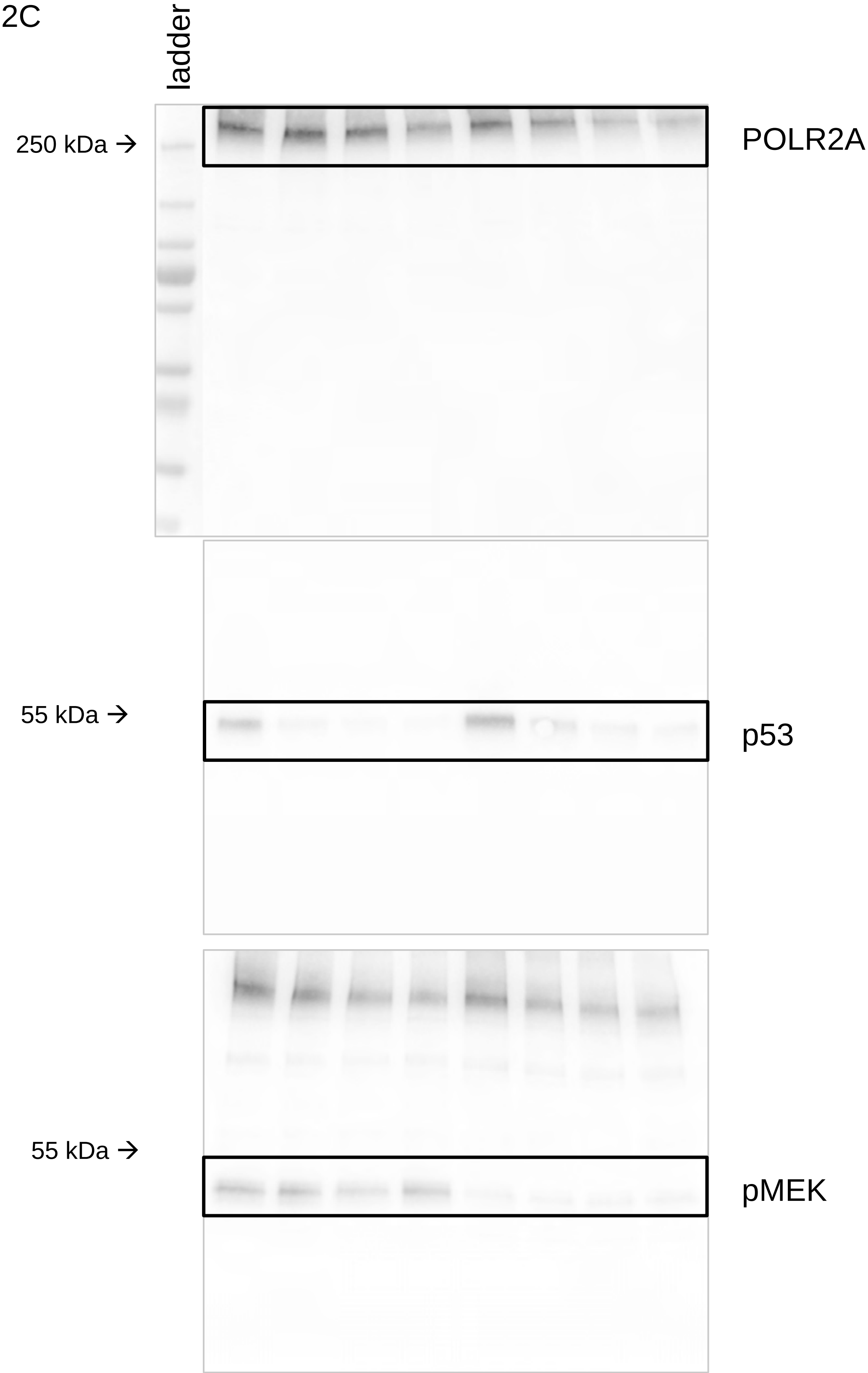

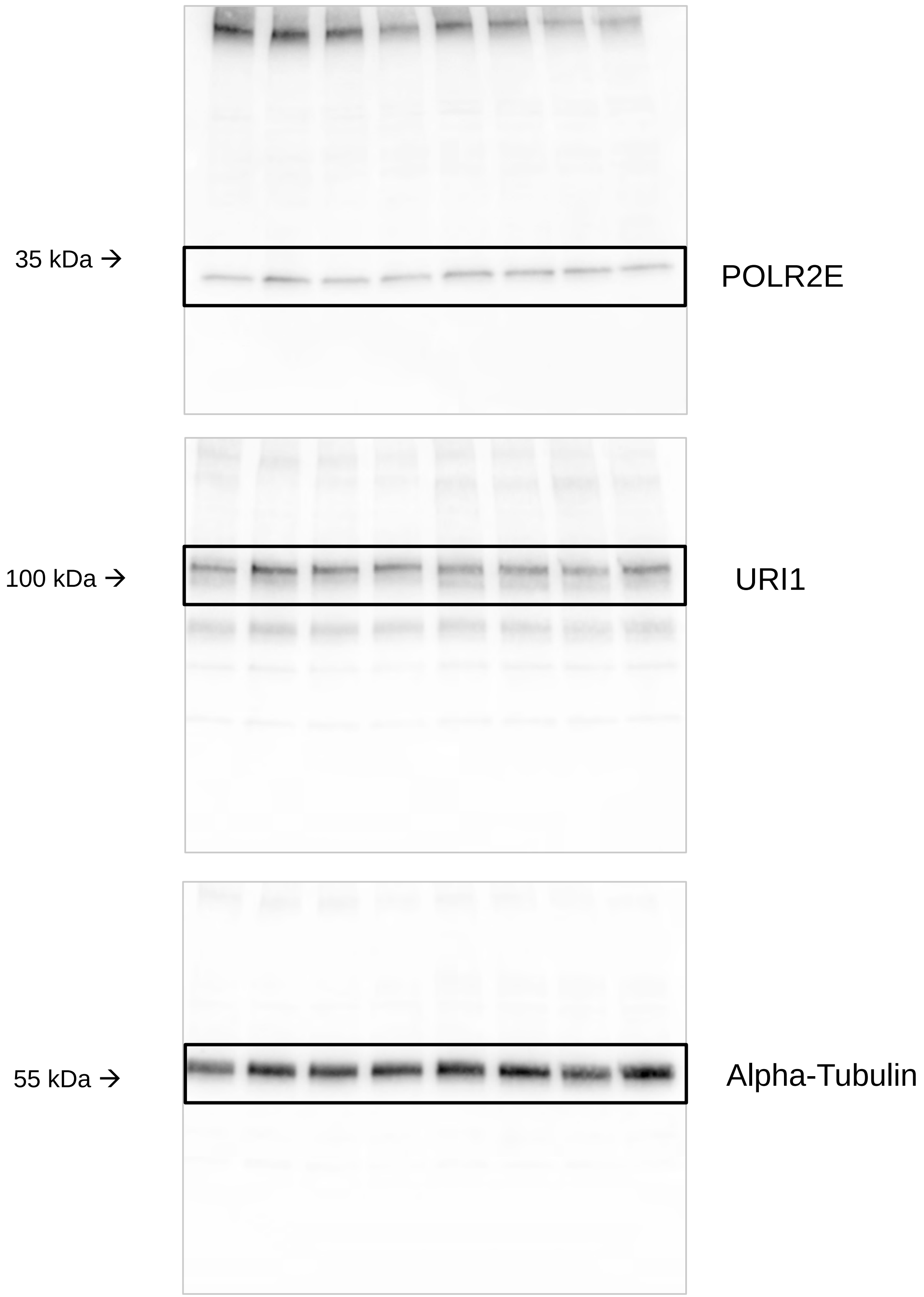
35 kDa →
POLR2E
100 kDa →
URI1
55 kDa →
Alpha-Tubulin

Figure 3A

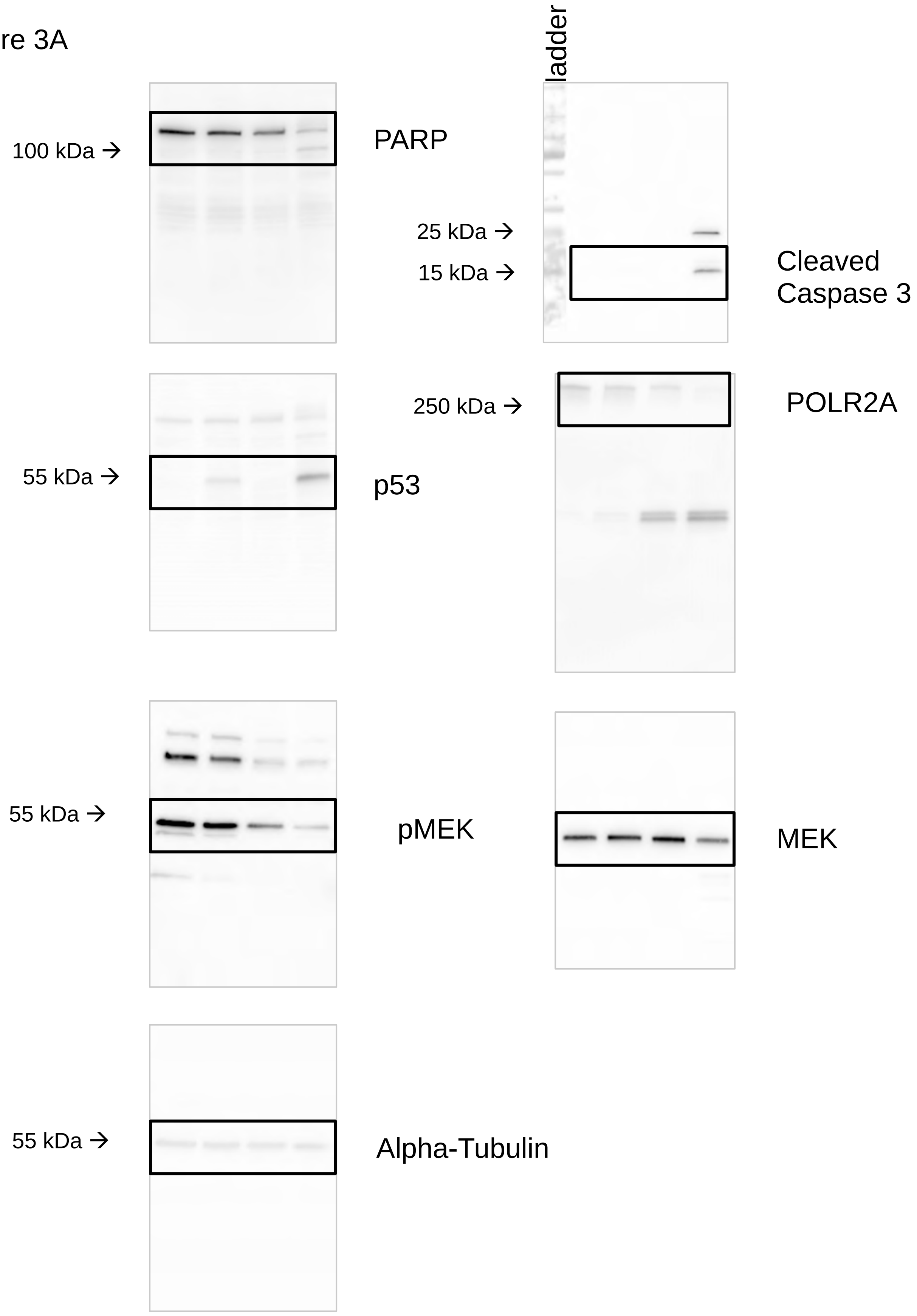

ladder
100 kDa →
PARP
25 kDa →
15 kDa →
Cleaved Caspase 3
250 kDa →
POLR2A
55 kDa →
p53
55 kDa →
pMEK
MEK
55 kDa →
Alpha-Tubulin

Figure 3B

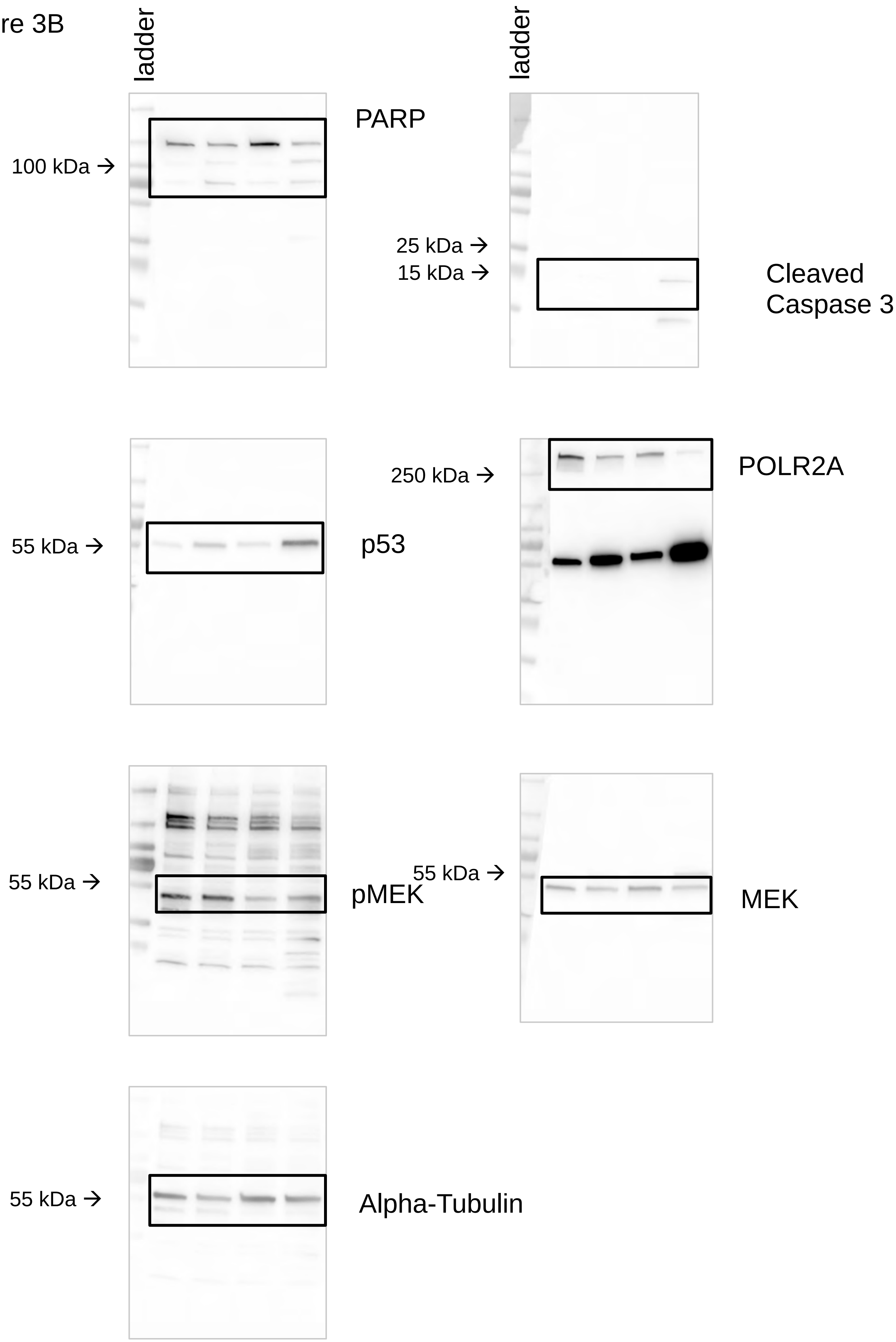
ladder
ladder
PARP
100 kDa →
25 kDa →
15 kDa →
Cleaved Caspase 3
55 kDa →
p53
250 kDa →
POLR2A
55 kDa →
pMEK
55 kDa →
MEK
55 kDa →
Alpha-Tubulin

Figure 3C

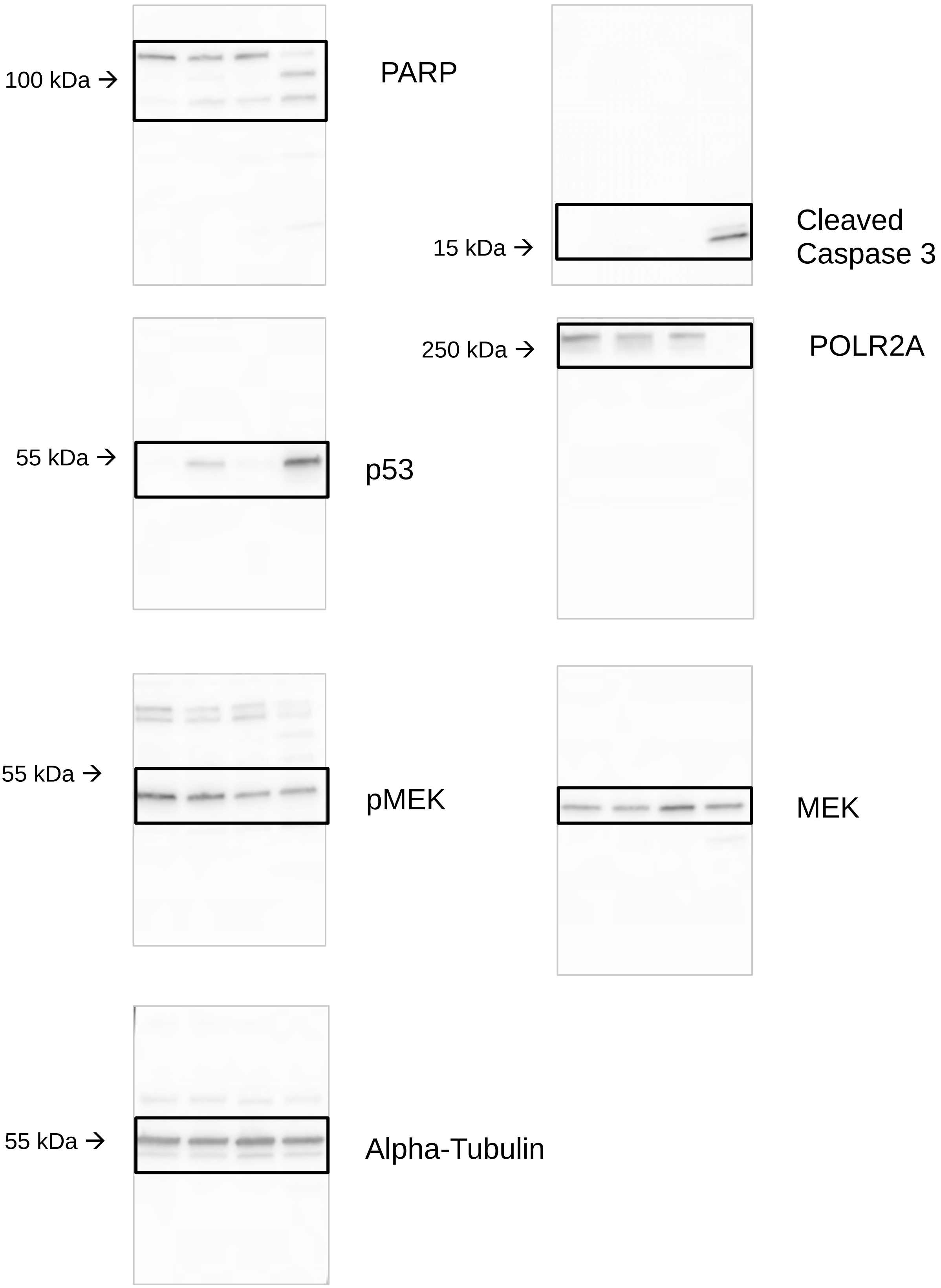

100 kDa →
PARP
15 kDa →
Cleaved Caspase 3
250 kDa →
POLR2A
55 kDa →
p53
55 kDa →
pMEK
MEK
55 kDa →
Alpha-Tubulin

Figure 4B

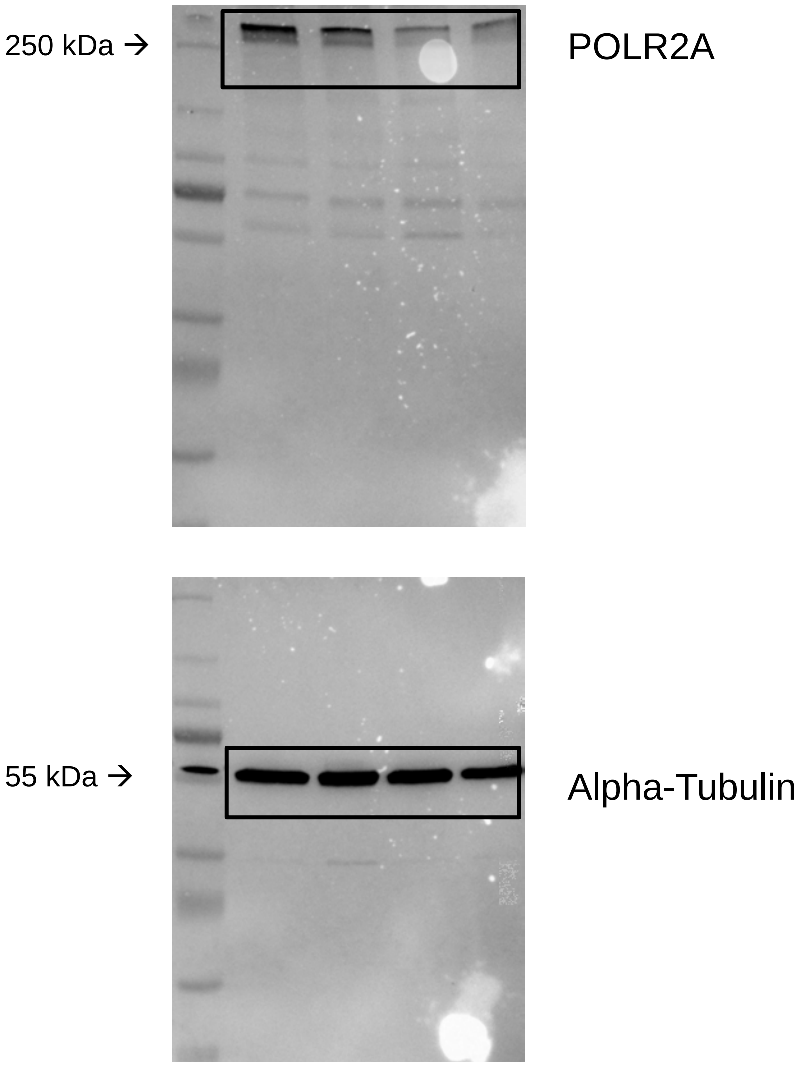

Figure S1A

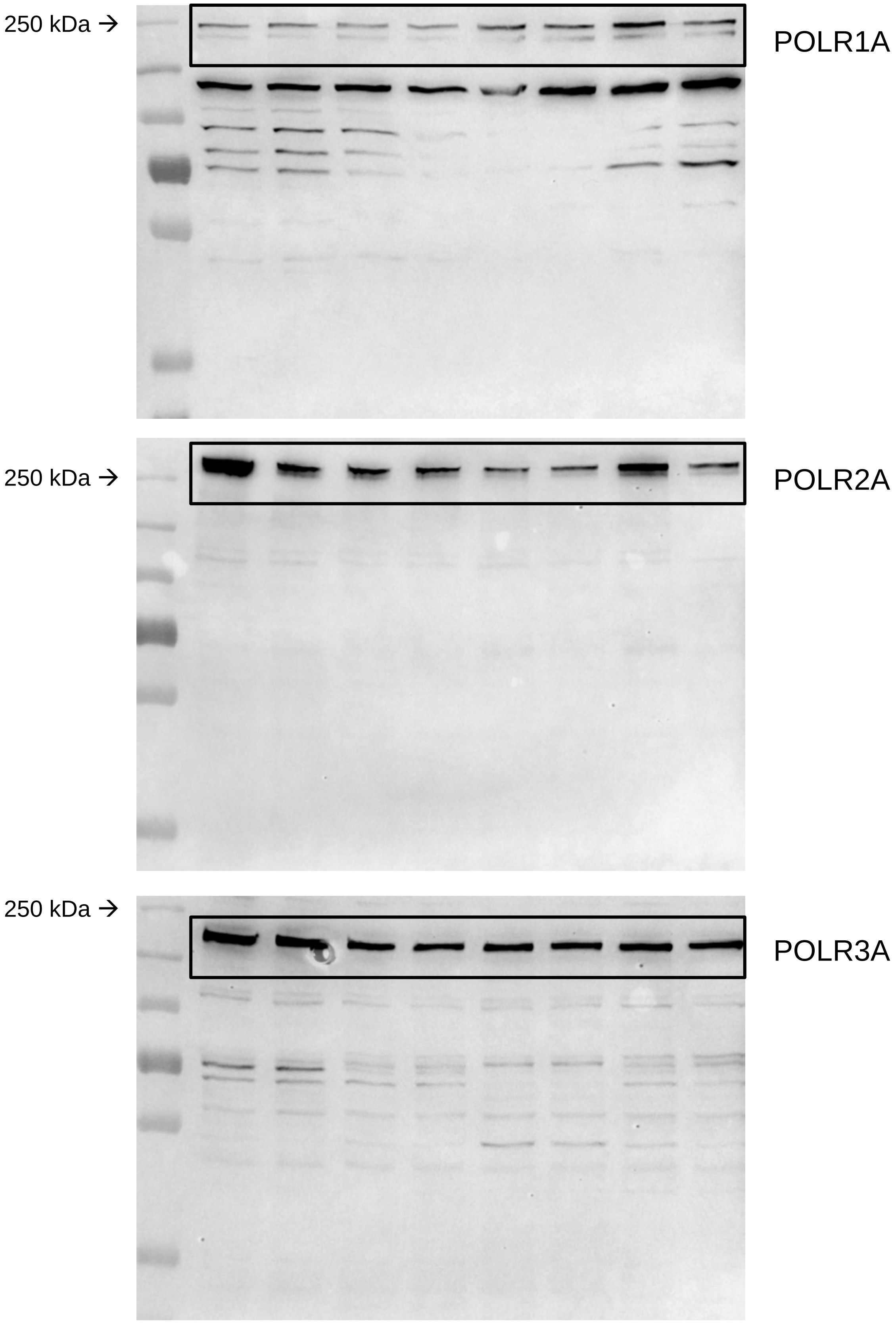

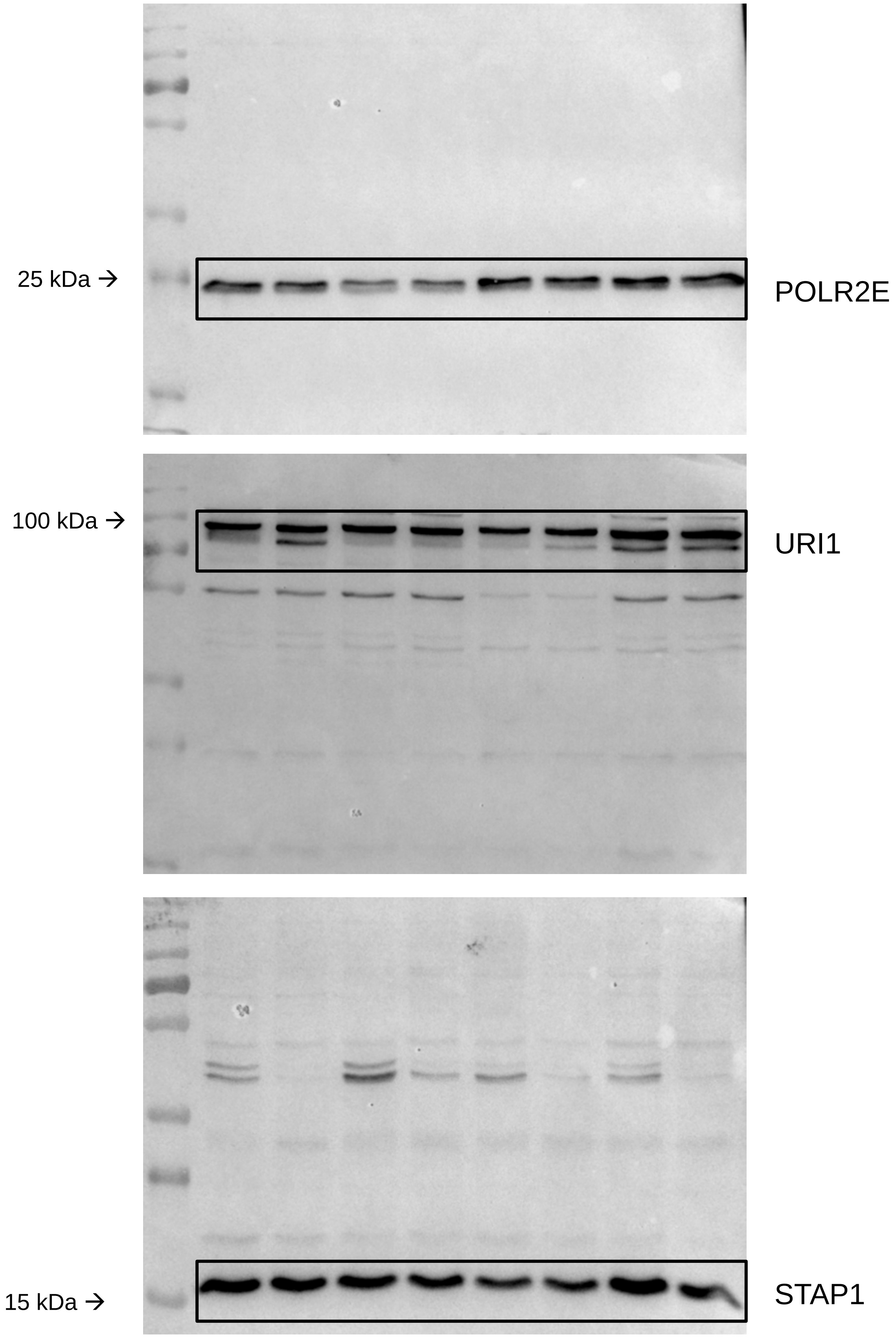

25 kDa →
POLR2E
100 kDa →
URI1
15 kDa →
STAP1

35 kDa →

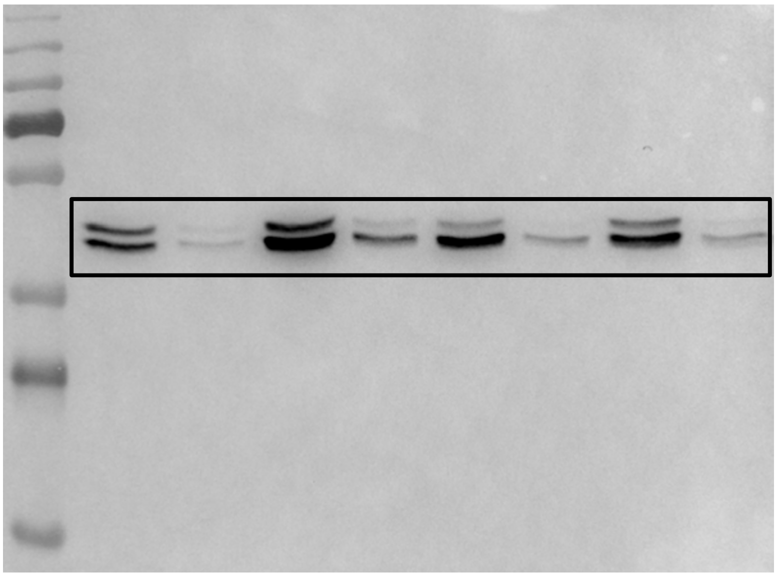

pERK

35kDa →

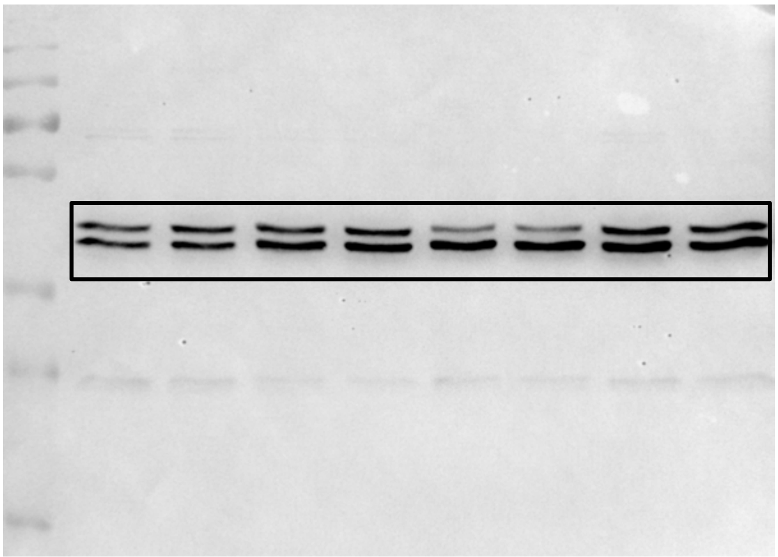

ERK

55 kDa →

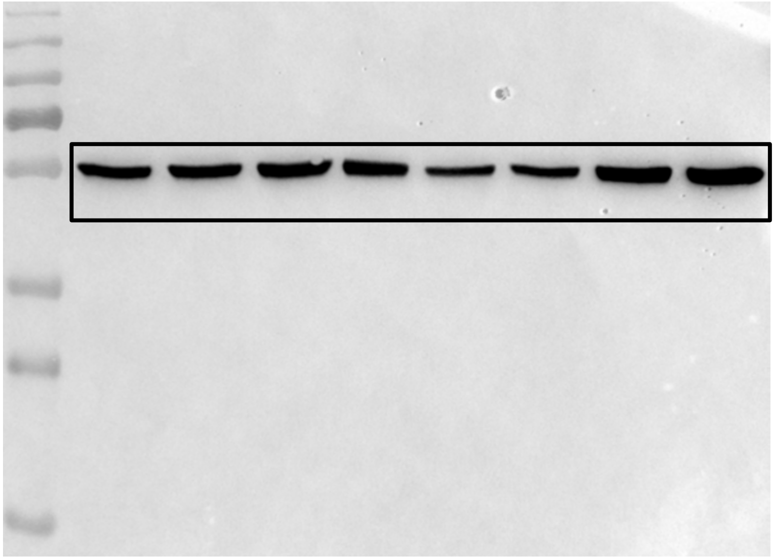

Alpha-tubulin

Figure S1B

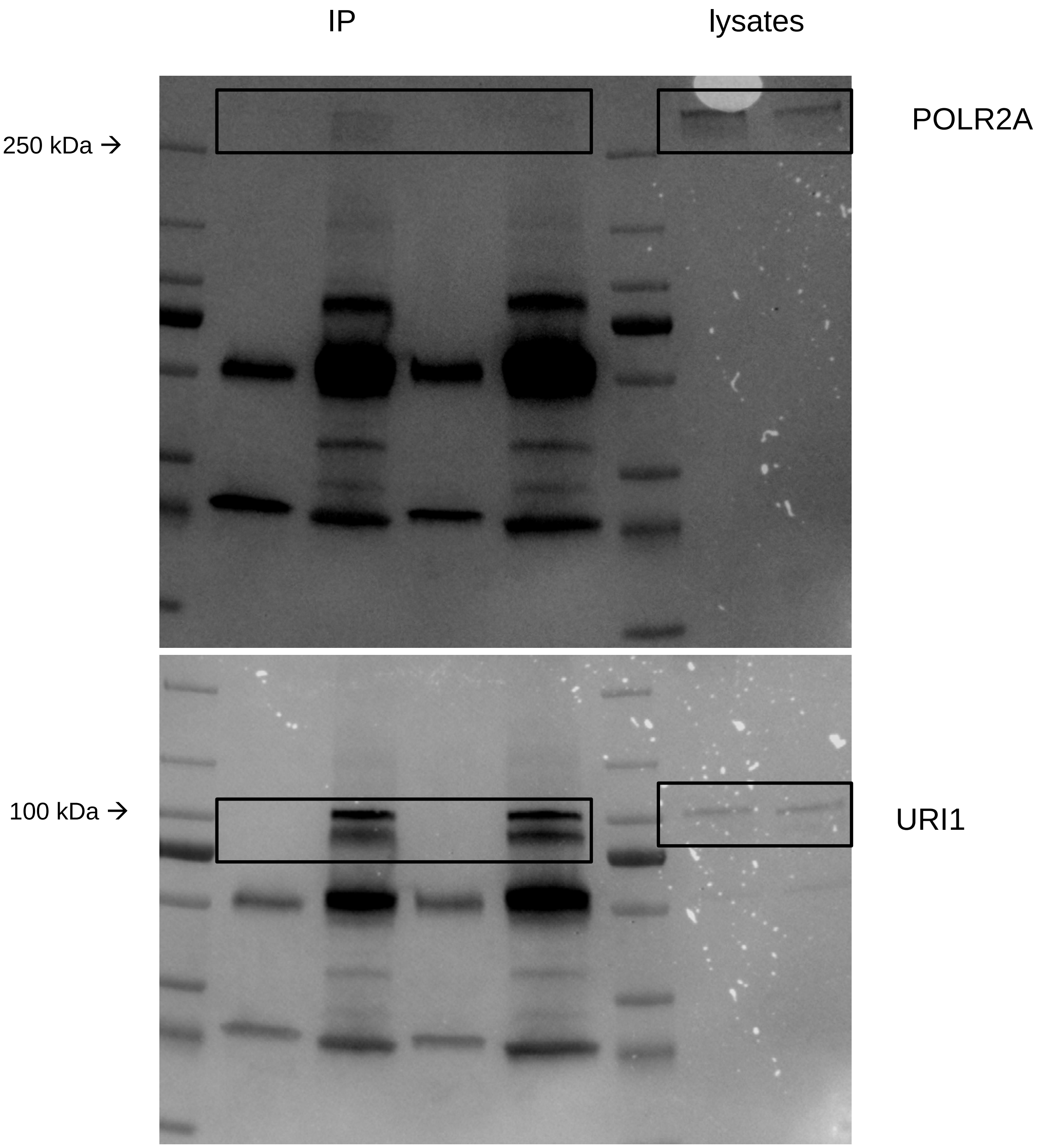

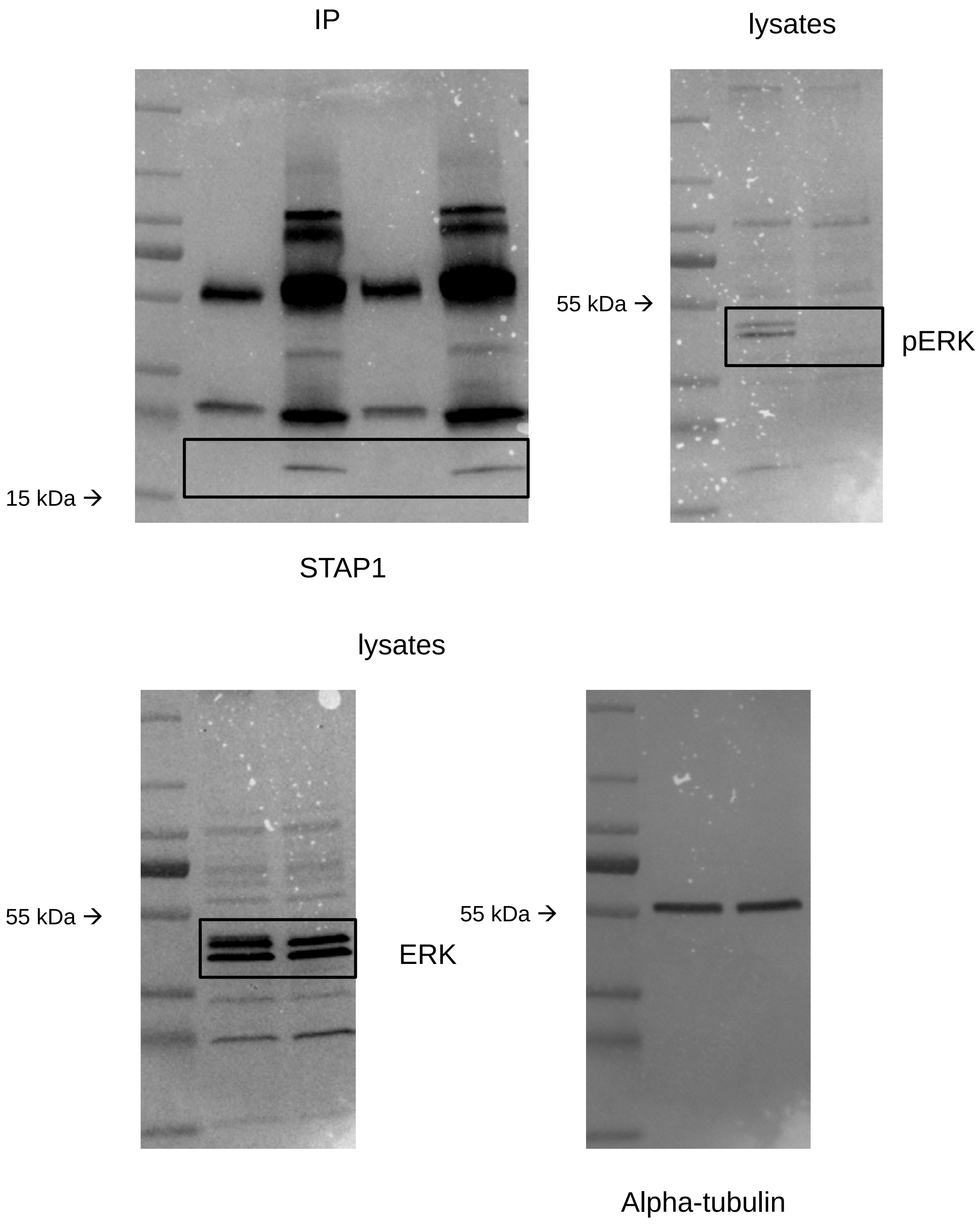
IP
lysates
55 kDa →
pERK
15 kDa →
STAP1
lysates
55 kDa →
ERK
55 kDa →
Alpha-tubulin

Figure S2A

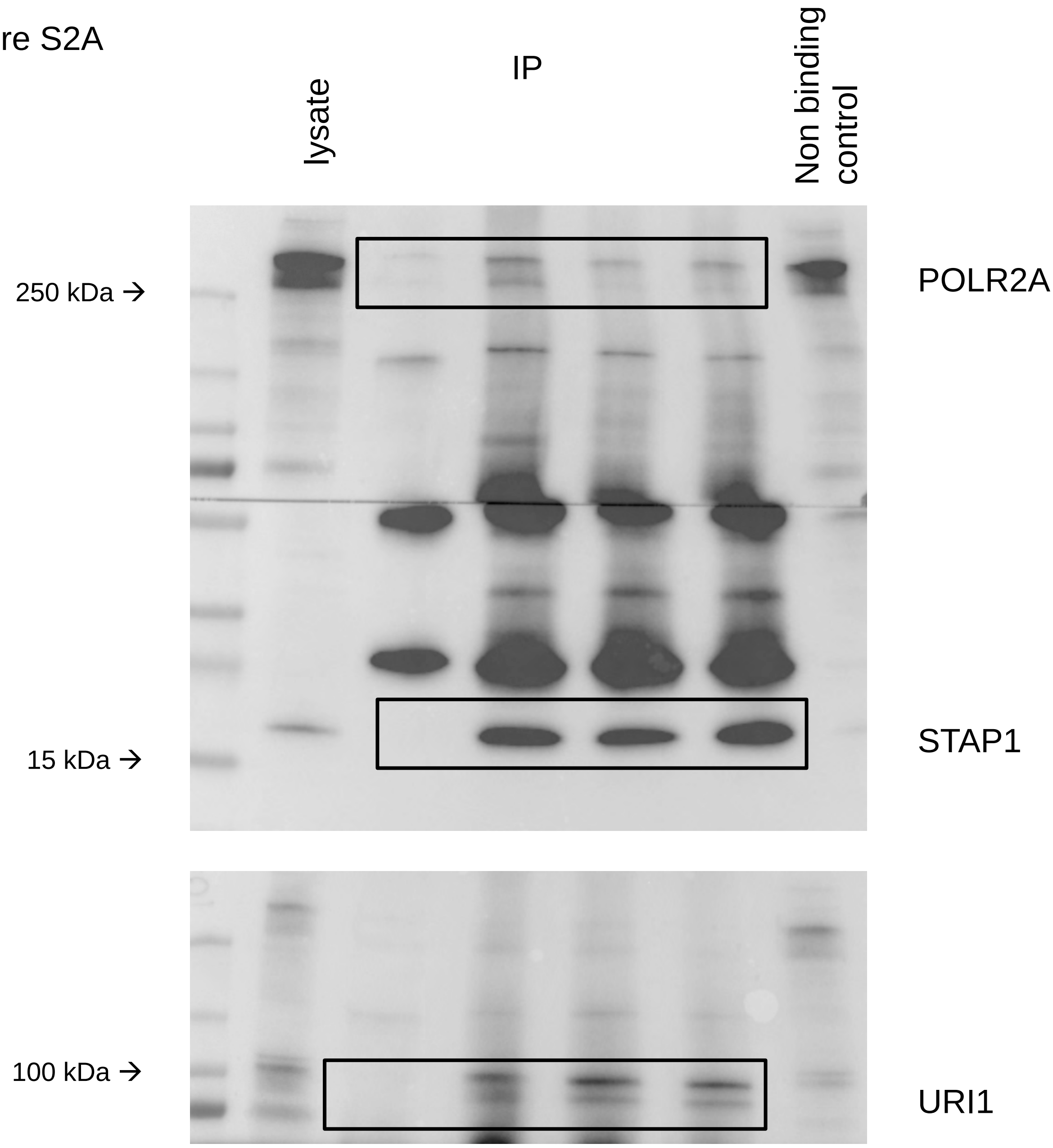
IP
lysate
Non binding control
250 kDa →
15 kDa →
100 kDa →
POLR2A
STAP1
URI1

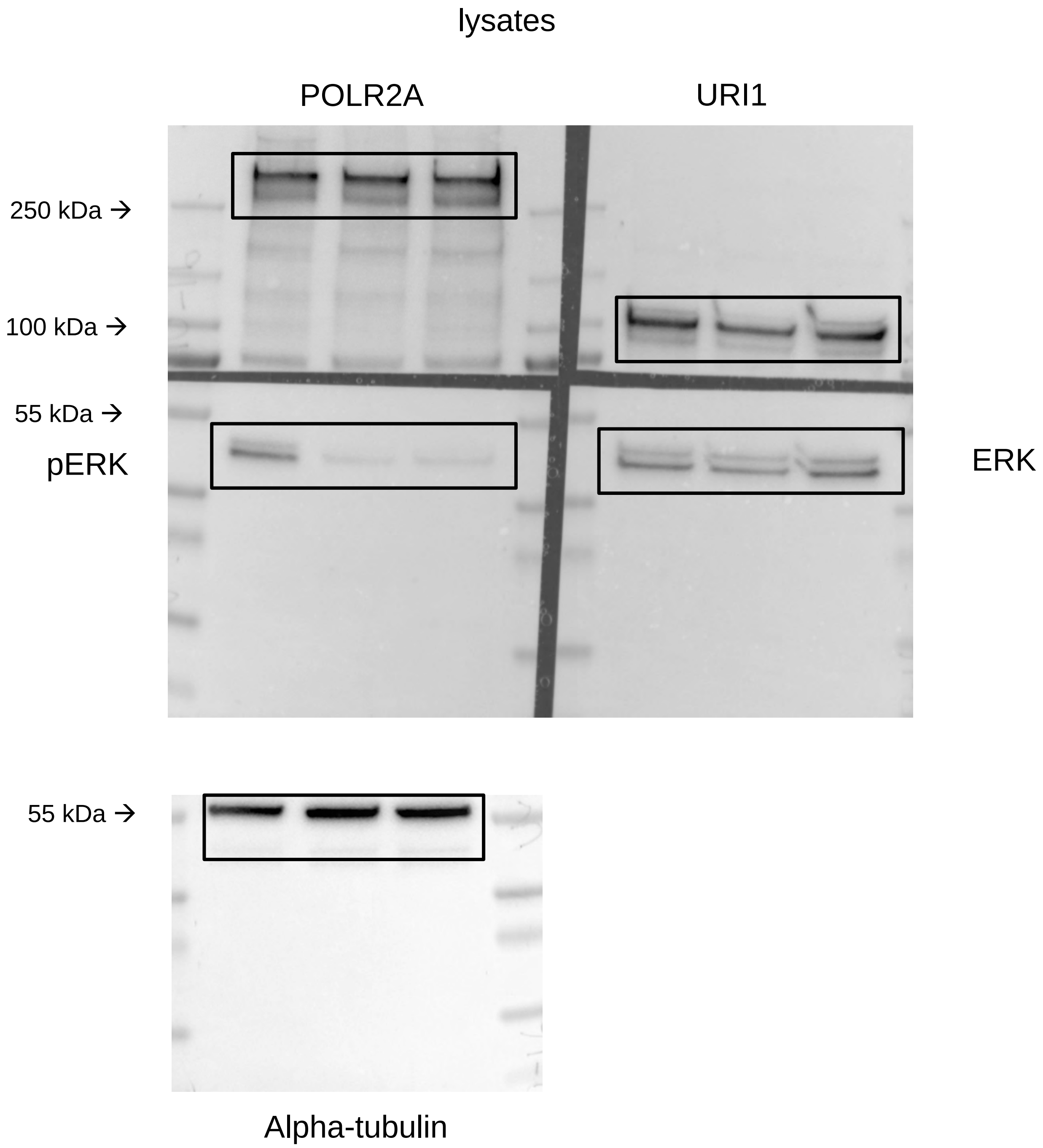
lysates
POLR2A
URI1
250 kDa →
100 kDa →
55 kDa →
pERK
ERK
55 kDa →
Alpha-tubulin
